# The temporal and spatial profiles of cell loss following experimental spinal cord injury: effect of antioxidant therapy on cell death and functional recovery

**DOI:** 10.1186/1471-2202-14-146

**Published:** 2013-11-18

**Authors:** Xiang Ling, Feng Bao, Hao Qian, Danxia Liu

**Affiliations:** 1 Department of Neurology, University of Texas Medical Branch, 301 University Blvd., Rt. 0881, Galveston, TX 77555-0881, USA; 2 Department of Biochemistry & Molecular Biology, University of Texas Medical Branch, 301 University Blvd., Rt. 0881, Galveston, TX 77555-0881, USA

**Keywords:** Antioxidant therapy, Apoptotic cell death, Behavioral test, Mn (III) tetrakis (4-benzoic acid) porphyrin, Nitro-L-arginine, Secondary spinal cord injury

## Abstract

**Background:**

Traumatic spinal cord injury (SCI)-induced overproduction of endogenous deleterious substances triggers secondary cell death to spread damage beyond the initial injury site. Substantial experimental evidence supports reactive species (RS) as important mediators of secondary cell death after SCI. This study established quantitative temporal and spatial profiles of cell loss, characterized apoptosis, and evaluated the effectiveness of a broad spectrum RS scavenger - Mn (III) tetrakis (4-benzoic acid) porphyrin (MnTBAP) and a combination of MnTBAP plus nitro-L-arginine to prevent cell loss and neurological dysfunction following contusion SCI to the rat spinal cord.

**Results:**

By counting the number of surviving cells in spinal cord sections removed at 1, 6, 12, 24, 48, 72 h and 1 week post-SCI and at 0 – 4 mm from the epicenter, the temporal and spatial profiles of motoneuron and glia loss were established. Motoneurons continued to disappear over a week and the losses decreased with increasing distance from the epicenter. Significant glia loss peaked at 24 to 48 h post-SCI, but only at sections 0–1.5 mm from the epicenter. Apoptosis of neurons, motoneurons and astrocytes was characterized morphologically by double immuno-staining with cell-specific markers and apoptosis indicators and confirmed by transmission electron microscopy. DNA laddering, *ELISA* quantitation and caspase-3 activation in the spinal cord tissue indicated more intense DNA fragments and greater caspase-3 activation in the epicenter than at 1 and 2 cm away from the epicenter or the sham-operated sections. Intraperitoneal treatment with MnTBAP + nitro-L-arginine significantly reduced motoneuron and cell loss and apoptosis in the gray and white matter compared with the vehicle-treated group. MnTBAP alone significantly reduced the number of apoptotic cells and improved functional recovery as evaluated by three behavioral tests.

**Conclusions:**

Our temporal and spatial profiles of cell loss provide data bases for determining the time and location for pharmacological intervention. Our demonstration that apoptosis follows SCI and that MnTBAP alone or MnTBAP + nitro-L-arginine significantly reduces apoptosis correlates SCI-induced apoptosis with RS overproduction. MnTBAP significantly improved functional recovery, which strongly supports the important role of antioxidant therapy in treating SCI and the candidacy of MnTBAP for such treatment.

## Background

Spinal cord injury (SCI) develops in two phases: primary injury and secondary injury. The initial mechanical injury not only directly kills cells and destroys various elements of the tissue, but also induces overproduction of endogenous deleterious substances, which trigger secondary damage processes that lead to secondary cell death, thereby spreading damage beyond the initial injury site [1,2]. Apoptosis is an important contributor to secondary cell death after SCI. Numerous studies have shown that neuronal and glial apoptosis follows SCI [3-6]. Cells die by necrosis or apoptosis depending on the types and concentrations of the triggering death signals. At the injury epicenter, the concentration of endogenous deleterious substances produced upon the initial trauma reaches a maximum, and then gradually declines with distance from the epicenter; in like manner, the type of cell death may also depend on the distance from the injury epicenter. Therefore, it is critical to explore the temporal and spatial profiles of cell death by necrosis and apoptosis for each type of cell to precisely define the time window and the damage area for therapeutic intervention.

Reactive species (RS), including free radicals and non-radical oxidants, are believed to contribute to secondary destruction after central nervous system (CNS) injury by oxidatively damaging the major cellular components proteins: DNA, and phospholipids [7-9]. Substantial experimental evidence supports RS as important mediators of secondary damage after SCI [10-13]. We and others have demonstrated that the levels of superoxide anion [6,14], hydrogen peroxide [15], hydroxyl radical [16], catalytic iron [17], nitric oxide (^•^NO) [18,19] and peroxynitrite [18,20], as well as the products of oxidation and nitration of proteins [6,18,21-23] and membrane lipid peroxidation (MLP) [24-26], all significantly increase following SCI. By administering the increased levels of RS into an un-injured rat spinal cord, we demonstrated that they caused oxidative damage to major cellular components [27,28], neuronal death by necrosis and apoptosis [29-31] and neurological dysfunction [29]. Our *in vivo* demonstration unequivocally links SCI-induced RS elevation to necrotic and apoptotic cell death and neurological dysfunction in SCI. Therefore, a broad spectrum scavenger of RS should more effectively reduce secondary cell death and the resulting neurological dysfunction than would agents with a single target.

Metalloporphyrins, a novel class of catalytic antioxidants, not only scavenge a wide range of RS such as superoxide anion, hydrogen peroxide, peroxynitrite and lipid peroxyl radicals [32], but also modulate RS-based redox signaling pathways [33]. The metalloporphyrin Mn (III) tetrakis (4-benzoic acid) porphyrin (MnTBAP) possesses both superoxide dismutase and catalase-like activity [34] and scavenges peroxynitrite [35]. It is also a potent inhibitor of MLP [36]. In the CNS, cerebroventricular injection of MnTBAP inhibited kainate-induced mitochondrial superoxide production, DNA oxidation and neuronal loss in the hippocampus of rat [37]. We demonstrated *in vivo* that MnTBAP reduced peroxynitrite-induced oxidation and nitration of proteins [27] and MLP [28] in the rat spinal cord. It prevented hydroxyl radical-induced necrotic and apoptotic cell death [31]. We recently demonstrated that intrathecal administration of MnTBAP reduced superoxide and hydrogen peroxide production, reduced oxidation and nitration of proteins and increased the number of surviving neurons, motoneurons, astrocytes and oligodendrocytes after SCI [38-40]. These results suggest that the catalytic antioxidant MnTBAP may be a potential agent for antioxidant therapy, owing to its cell permeability, low toxicity and broad scavenging of RS.

However, MnTBAP was reported poorly able to penetrate the blood–brain barrier [41], so it does not seem to be a favorable candidate for antioxidant therapy for CNS injury and degenerative disorders. We compared the penetrating ability of MnTBAP to methylprednisolone (MP), the only drug used clinically to treat SCI. We found that, despite the much lower penetration of the blood-spinal cord barrier (BSB) by MnTBAP compared with MP, its higher stability allows a lower dose of MnTBAP to produce a higher concentration in the CSF than does higher doses of MP [42]. Moreover, we demonstrated that MnTBAP (10 mg/kg) given intraperitoneally (i.p.) increased the number of neurons and attenuated the number of apoptotic neurons after SCI [43]. Treatment with this dose of MnTBAP (i.p.) more effectively improved the functional recovery after SCI than did the standard MP regimen [40]. These results suggest that MnTBAP indeed crossed the BSB and reached the appropriate targets, perhaps in part, because the injury disrupted the BSB to allow MnTBAP to pass through. Therefore, MnTBAP warrants further examination of its antioxidative efficacy.

In our previous study, we established the temporal and spatial profile of neuron death, the temporal and spatial profiles of the appearance of apoptotic cells in the gray and white matter and the temporal and spatial profiles of neuronal apoptosis after SCI [43]. The goals of the present study were the following. 1) To established the temporal and spatial profiles of glial cell loss in the white matter and motoneuron loss in the gray matter for up to one week following severe injury to the rat lumbar enlargement. 2) To characterize SCI-induced apoptosis of neurons, motoneurons and glial cells morphologically and biochemically. 3) To evaluate the efficacy of cell protection by MnTBAP and a combination of MnTBAP plus nitro-L-arginine (L-NA) ― a non-selective nitric oxide synthase (NOS) inhibitor. It was reported that MnTBAP is not a scavenger of ^•^NO [44]. However, the toxicity of ^•^NO occurs when it reacts with overproduced superoxide to form peroxynitrite - a strong oxidant, whereas MnTBAP scavenges peroxynitrite. Therefore, both MnTBAP alone and the combination of MnTBAP + L-NA will be tested for their protection against cell loss following SCI. 4) To test the ability of MnTBAP to improve functional recovery after SCI by behavioral tests. Accomplishment of these goals will provide a foundation for determining the optimal time and location to test the therapeutic potential of pharmaceutical agents to prevent cell death and apoptosis and add new information for exploring the therapeutic potential of the catalyst antioxidant MnTBAP in SCI treatment.

## Methods

All animal experimental procedures were approved by the University of Texas Medical Branch Animal Care and Use Committee and were in accord with the NIH *Guide for* the *Care and Use of Laboratory Animals*.

### Establishing cell loss profiles and characterizing cellular apoptosis morphologically following SCI

#### Animal preparation, spinal cord injury and drug administration

To obtain the temporal and spatial profiles of cell loss after SCI, male Sprague–Dawley rats (250–300 g) were randomly divided into two groups: injured and sham operated. Animals were anesthetized with pentobarbital (50 mg/kg, i.p.). When the rat was fully anesthetized, a laminectomy was performed on vertebra T13 and the dura was kept intact. Care was taken not to injure the cord. Then the animal was clamped in a frame by attachments to its dorsal vertebral processes. The cord was injured severely by dropping a weight (10 g) through a guide tube 5 cm onto the cord (50 g.cm) according to the impact method originally devised by Allen [45] and modified by us. The weight was fitted with a Teflon tip with an area of 10 mm^2^. After injury, the incision was surgically repaired and the rats were housed in a temperature- and humidity-controlled room with a 12 h light/dark cycle. Animals had free access to food and water. The procedures for anesthesia, surgery and impact injury are described in detail in our previous publications [14-18,21]. The only difference in animal preparation between the sham-operated control and the injured group was that no impact injury was performed in the sham rats.

To test the protection against cell death by the combination of MnTBAP (Calbiochem, San Diego, CA, USA) and L-NA, the animal was injured as above, then MnTBAP (10 mg/kg) plus L-NA (1 mg/kg) dissolved in saline was given i.p. at 15 min pre- and 6 h post-SCI. To obtain the spatial profiles of MnTBAP protection against apoptotic cell death, MnTBAP alone (10 mg/kg in saline) was administered by i.p. injection as described above. In the vehicle control group, animals were injured and saline was administered as in the treated group.

#### Spinal cord tissue processing for morphological examination

To establish the temporal and spatial profiles of motoneuron and glial cell loss, the injured cords were harvested at 1, 6, 12, 24, 48, 72 h and 1 week post-SCI as the same time points as for establishing the temporal and spatial profile of neuron loss [43], so the profiles of different cell types can be compared. At the time of cord removal, the animals were re-anesthetized with pentobarbital (50 mg/kg), perfused intracardially with 0.9% saline, followed by 4% paraformaldehyde in 0.1 M phosphate-buffered saline (PBS, pH 7.2-7.4) while the animal was still deeply anesthetized. A second laminectomy was performed on vertebrae T11-L3 to extend the exposed cord, so that segments approximately 1.5 cm long centered at the injury site were removed and fixed in the same fixative overnight and embedded in paraffin. Rats that served as sham-operated controls were similarly perfused at 12 h post-operation. Serial sections (10 μm thick) were cut and mounted on glass slides coated with poly-L-lysine.

The sections at 0.1 mm (10 sections) intervals from every cord were deparaffinized and stained histochemically with cresyl violet (CV) as describe in our previous publications [31]. The CV-stained sections were observed under a light microscope to locate the epicenter (with the lowest cell density). After the epicenter was determined, the spinal sections at 0, 0.5, 1.0, 1.5, 2.0, 2.5, 3.0, 3.5 and 4.0 mm caudal from the epicenter at each time point post-SCI were CV-stained to obtain the temporal and spatial profiles of cell death.

From our temporal and spatial profiles, we found that severe neuron [43] and motoneuron loss near the epicenter starting at 12 and 24 h post-SCI and gradually spread to longer distance over time. Our temporal and spatial profiles of apoptosis published previously [43], indicated that the peak time of TUNEL-positive cell appearances are from 12 to 48 h post-SCI. Therefore, 12 to 48 h post-SCI is a reasonable time frame in which to evaluate the efficiency of treatment to reduce cell death and apoptosis. The rostral sections removed at 12 h post-SCI at 0–4.0 mm from the epicenter were also CV-stained to be used as vehicle controls to establish the spatial profile of MnTBAP + L-NA protection against cell death and for terminal deoxynucleotidyl transferase (TdT)-mediated deoxyuridine triphosphate-(dUTP)-biotin nick end labeling (TUNEL) staining to evaluate the protection of the combined treatment against apoptosis. The rostral sections obtained at 24 h post-SCI at 0, 1.0, 2.0 and 4.0 mm from the epicenter were used for TUNEL staining as vehicle-treated group to evaluate MnTBAP protection against apoptotic cell death and for characterization of apoptosis. To match with the vehicle-treated groups, the injured spinal cord in the MnTBAP + L-NA-treated rats was removed at 12 h post-SCI and the injured cord in the MnTBAP-treated rats was removed at 24 h post-SCI.

#### TUNEL staining and TUNEL plus immunohistochemical or immuno-fluorescence double staining to characterize apoptosis in different types of cells in injured spinal sections

DNA fragmentation in the nuclei is an indicator of apoptosis. Fragmented DNA in the nuclei of cells was characterized by TUNEL staining using an ApopoTag peroxidase *in situ* apoptosis detection kit according to the manufacturer’s instructions. The TUNEL staining and visualization of spinal cord sections were performed according to procedures we previously reported [30,31,43]. The only difference was that, in the present study, the TUNEL detecting kit was purchased from Millipore (Billerica, MA, USA). Nonspecific labeling was investigated by using water or equilibration buffer to substitute for the volume of TdT in the TUNEL reaction mixture.

To characterize apoptosis in different types of cells, TUNEL plus immunohistochemical double staining was performed. The spinal cord sections were first stained with TUNEL, then stained with antibodies specific to different types of cells. Following TUNEL staining, the sections were washed in three changes of PBS for 5 min each, blocked with 5% goat serum and incubated with: a mouse monoclonal antibody directed against neuron specific enolase (NSE, 1:100, DAKO, Carpinteria, CA, USA) to stain neurons; a rabbit polyclonal antibody directed against choline acetyltransferase (ChAT, 1:500, Abcam, Cambridge, UK) to stain motoneurons; or a monoclonal antibody against glial fibrillary acidic protein (GFAP, 1:200, Sigma, St. Louis, MO, USA) to stain astrocytes. After washing in PBS, the sections were incubated with biotinylated goat anti-rabbit-IgG (1:200, Sigma, St. Louis, MO, USA) or goat anti-mouse-IgG (1:200, Sigma, St. Louis, MO, USA) followed by a solution of an avidin-biotin-horseradish peroxidase complex (1:200, Vector Laboratories, Inc., Burlingame, CA, USA). The sections were then visualized with SG Substrate Kit (Vector Laboratories, Inc., Burlingame, CA, USA), washed in water, dehydrated, cleaned and coverslipped with Permount as described in our previous report [30,31,43]. Negative controls were similarly stained without the primary antibody.

Motoneurons were also characterized with TUNEL and immuno-fluorescence double staining. The TUNEL staining was performed similarly as above, but using the ApopTag plus fluorescein *In Situ* apoptosis detection kit (Chemicon Intl. Temecula, CA, USA) following the manufacturer’s instruction. After TUNEL staining, the sections were immuno-stained as in the above procedures with the primary antibody, mouse anti-ChAT (1:100, ABcam, Cambridge, MA, USA). After washing in PBS for 3 times, a secondary antibody goat anti-mouse Alexa Flour 594 (Invitrogen, Carlsbad, CA, USA) was added to the sections and the sections were incubated for 1 h at room temperature. The sections were washed in PBS and coversliped with anti-fade solution (Invitrogen) and kept in the dark. Negative controls were similarly stained without the primary antibody.

#### Active caspase-3 and cellular marker immuno-fluorescence double staining

Caspase-3 activation is another indicator of apoptosis. To characterize apoptosis in different types of cells, double immuno-fluorescence staining with antibodies against active caspase-3 and specific cellular markers was performed. The procedures for immuno-fluorescence staining were similar to those described above. The deparaffinized spinal cord sections were rinsed in PBS and placed in 5% goat serum in PBS for 30 min, then incubated with the primary antibody: a monoclonal antibody anti-NSE (1:100, DAKO, Carpinteria, CA, USA) or a monoclonal antibody anti-GFAP (1:200, Sigma, St. Louis, MO, USA) to stain neurons or astrocytes, respectively. For immunohistochemical staining of active caspase-3, the immuno-stained sections were incubated with the secondary antibody, a polyclonal antibody anti-p20 active fragment of caspase-3 (1:200, R&D systems, Inc. Minneapolis, MN) in PBS with 0.2% Triton X-100 and 0.1% bovine serum albumin overnight at 4°C. For visualization, after washing in PBS, the sections were incubated with fluorescein isothiocyanate (FITC)-labeled goat anti-mouse IgG (1:100, Sigma, St. Louis, MO, USA) and crystalline tetramethylrhodamine isothiocyanate (TRITC)-labeled goat anti-rabbit IgG (1:100, Sigma, St. Louis, MO, USA) in PBS with 0.2% Triton X-100 and 0.1% BSA for 1 hour in the dark to visualize cells and active caspase-3, respectively. The slides were coverslipped with PBS/glycerol and stored at 4°C after washing with PBS in the dark. The FITC-labeled neurons or astrocytes display green and the TRITC-labeled activated caspase-3 displays red fluorescence. Negative controls were similarly stained without the primary antibody.

#### Confirmation of apoptosis by transmission electron microscopy (TEM)

For TEM examination of neuronal and glial apoptosis, the animal preparation and spinal cord injury were done as described above. At 24 h post-SCI, the rat was perfused intracardially with 0.9% saline, followed by 4% paraformaldehyde with 2.0% glutaraldehyde in 0.01 M PBS (pH 7.4). After perfusion, the spinal cord was further exposed and a 1.5 cm segment centered at the injury site was removed as described above and fixed in the same fixative overnight. Then, the segment was fixed with 4% osmium tetroxide in 0.01 M PBS for 2 h, dehydrated and embedded in epoxy resin. Semi-thin (1 μm) plastic sections at 2-2.5 mm from the epicenter were cut and stained with 1% toluidine blue to screen for apoptotic neurons under the light microscope. Ultrathin sections (in the Rexed lamina VIII, gold interference color) were cut with an ultramicrotome, stained with uranyl acetate and lead citrate, then examined using a Hitachi H600 electron microscope (Tokyo, Japan).

#### Quantitation of cell loss and apoptosis by counting surviving or TUNEL-positive cells in spinal cord sections

Surviving cells in the CV-stained sections or TUNEL-positive cells in the TUNEL-stained sections were counted in an area of the bottom left and bottom right quarter of the gray matter of the cord. Quarters were defined by horizontal and vertical crossed lines drawn through the central canal in the center of the sections [18,38-40,43]. This area of gray matter includes motoneurons, middle- and small-sized neurons, and glial cells. Neurons in the CV-stained sections were identified by the presence of bright nuclei with nucleolus and enrichment in Nissl bodies. Neurons with soma diameters greater than 25 μm were identified and counted as motor neurons [46]. Glial cells in the CV-stained sections were counted in 250 × 250 μm^2^ areas of the left and right ventromedial white matter. TUNEL-positive cells were identified by dark nuclei and were counted in TUNEL-stained sections. The cells in the left or right quarters of each section were counted 3 times and the counts averaged. Then, the average counts of the left and right quarters in each section were averaged to obtain the final counts for that section. Three adjacent sections were counted and the counts averaged to obtain the final counts for that distance. To avoid bias, the person processing the cord into sections and the person counting the cells in the sections were blinded. This counting method has been validated by our previous publications [18,38-40,43] and by those of many others.

### Biochemical characterization and quantification of apoptosis in spinal cord tissues following SCI

#### Animal preparation and tissue processing

The animal preparation and impact injury were the same as described above. At 48 h post-SCI (or post-laminectomy for sham controls), the animal was re-anesthetized and the cord was further exposed from the vertebrae T10 to L5 by a second laminectomy. A cavity was formed by pulling up the skin around the exposed cord and the spinal cord was frozen in situ by pouring liquid nitrogen (−196°C) into the cavity as described previously [21]. The frozen tissue was cut into three sections of equal length (1 cm each) with section 1 centered at the epicenter and sections 2 and 3 centered at 1 and 2 cm caudal to the epicenter. The sections were immediately stored in a −80°C freezer for characterization of specific laddering of DNA fragments by agarose gel electrophoresis and for quantitation by enzyme-linked immuno-sorbent assay (*ELISA*). Since caspase-3 activates as early as 4 h post-SCI [6], to analyze caspase-3 activity, the frozen tissue was removed at 4 h post-SCI. The cord was cut into 2 sections (1 cm each) with section 1 centered at the epicenter and section 2 centered 1 cm from the epicenter.

#### DNA laddering by agarose gel electrophoresis

DNA fragmentation was characterized by radio-end labeling using [^32^P]dCTP and agarose gel electrophoresis following procedures reported [47]. Briefly, the spinal cord sections were removed and divided at 48 h post-SCI (5–10 mg wet weight), then gently disrupted with a hand-operated glass/glass Dounce homogenizer in lysis buffer (5 mM Tris–HCl, pH 7.4, containing 0.5% Triton X-100, 1:15 w:v), left on ice for 20 min, centrifuged for 30 min at 4°C at 15,000 g to remove the nuclei and cellular debris, then extracted with phenol:chloroform:isoamyl alcohol (25:24:1). After centrifugation, DNA in the aqueous phase was precipitated using ethanol. The DNA pellet was resuspended in 20 μl of Tris-EDTA buffer (pH 8.0) and treated with 20 μg/ml RNase A for 1 h at 37°C to digest the RNA. After quantitation, 2 μg of DNA from each sample was labeled with [^32^P]dCTP (50 μCi) by the standard method involving the Klenow fragment of DNA polymerase I. Samples were then re-extracted as above to remove unincorporated nucleotides, resuspended in DNA gel loading buffer and loaded onto a 2% agarose gel. After electrophoresis, the gel was dried under vacuum and exposed for 24 h to autoradiographic film.

#### Quantitation of DNA fragmentation by ELISA

Frozen spinal cord removed at 48 h post-injury was homogenized in lysis buffer containing 8 M urea, 0.24 M sodium phosphate, 1% sodium dodecyl sulfate and 10 mM EDTA, pH 6.8 using a 20:1 solution to tissue ratio (v:w). The homogenates were extracted with an equal volume of phenol-chloroform-isoamyl alcohol (25:24:1) saturated with the homogenization buffer. The resulting emulsion was separated into two phases by centrifugation at 4,500 *g* for 3 min in an Eppendorf centrifuge. The aqueous phase above the organic phase was removed for DNA analysis as reported originally by Beland and coworkers [48] and slightly modified by us [21]. Aliquots (10 μl) of each sample were used to quantitate the nucleosome-associated apoptotic DNA fragments by *ELISA* using a commercial cell death detection kit (Boehringer Mannheim, Indianapolis, IN, USA) which detects histone-associated DNA fragments enriched in the cytoplasm of a dying cell. The analysis was performed in duplicate according to the manufacturer’s instructions.

#### Analysis of caspase-3-like protease activity

Caspase-3-like protease activity was measured according to the reported procedure [49,50]. Briefly, approximately 40 mg of spinal cord tissue was homogenized in a 25 mM 4-(2-hydroxyethyl)-1-piperazineethanesulfonic acid (HEPES) buffer (pH 7.5) containing 1 mM ethylene glycol-bis (β-aminoethyl ether) N,N,N’,N’-tetraacetic acid, 5 mM MgCl_2_, 1 mM phenylmethyl-sulphonyl fluoride and 1 μg/ml leupetin and aprotinin, then centrifuged at 12,000 g for 15 min. The protein concentration in the supernatant was detected using the Bio-Rad Protein Assay kit (Bio-Rad Laboratories, Hercules, CA, USA). Then, 75 μg protein was incubated with synthetic fluorescent substrates benzyloxycarbonyl-Asp-Glu-Val-Asp-7-amino-4-trifluoromethylcoumarin (Z-DEVD-AFC, Enzyme Systems Products, Livermore, CA, USA) to assay the caspase-3-like protease activity at a concentration of 50 μM in 300 μl of 0.1 M HEPES buffer (pH 7.4) at 37°C for 1 h. The HEPES buffer contains 2 mM dithiothreitol, 0.1% CHAPS (3-[(3-cholamidopropyl) dimethylammonio]-1-propane-sulfonate) and 10% sucrose. DEVD-AFC cleavage was measured using a spectrafluorimetric detector (Shimadzu RF-10A, Shimadzu Europa GmbH, Duisburg, Germany) at an excitation wavelength of 400 nm and an emission wavelength of 505 nm. The fluorescence intensity was calibrated with a standard concentration of AFC and expressed in pmol per minute per mg of protein.

### Behavioral assessments

The protective effect of MnTBAP on the functional recovery after SCI was assessed by the Basso-Beattie-Bresnahan locomotor rating scale (BBB test), the inclined plane test, and the beam walk test for 9 weeks. SCI causes hind limb paralysis and inability of the rat to support its body weight, so the scrotum of male rats touched and dragged around the bottom of cage, increasing the incidence of infection. Therefore, most publications using behavioral tests to study SCI use female rats. This study examined the efficacy of MnTBAP to attenuate neurological deficits in three groups of female rats (200–225 g): injured groups treated with saline or MnTBAP (n = 8 for injured groups) and sham-operated controls (n = 4).

The injuries for behavioral tests to evaluate functional recovery after SCI are performed on T9 or T10 by using the standard New York University (NYU) weight drop device [40,51,52] or the Infinite Horizons (IH, Lexington, KY) device [53-55] in many publications. To explore the therapeutic potential of MnTBAP in treating SCI by behavioral tests, in the present study, the cord was injured on vertebra T10 using the NYU device, so that the results can be compared with other publications using behavioral tests.

#### Animal preparation, impact injury and drug administration

The rats were given 0.1 mg/kg buprenorphine subcutaneously (s.c.) 15 min prior to anesthesia, then anesthetized with pentobarbital (50 mg/kg, i.p.). After a laminectomy, the exposed cords were injured on vertebra T10 by a 10 g rod dropping 1.25 cm onto the exposed cord (12.5 g.cm force) using NYU device. Then, the muscle and skin were sutured in layers and 10 ml saline was injected (i.p.) to prevent dehydration. The body temperature of the rat was maintained using a feedback-controlled heating pad during surgery and until the animal awoke from surgery. MnTBAP (10 mg/kg) or saline was given by i.p. injection 15 min pre- and 4 h post-SCI for behavioral tests. In the sham-operated control group, animals underwent the same surgical procedures but no weight drop injury was performed. These rats were housed in a temperature- and humidity-controlled room with a 12 h light/dark cycle. In the first week following SCI, the bladder was emptied manually until the voiding reflex was recovered; buprenorphrine (0.5 mg/kg, s.c.) was given post-injury to minimize the impairment or discomfort of injured rats. The body weight of the animal was monitored during behavioral tests. If the loss of body weight was more than 20% pre-injury weight, the animal was terminated. Since only the hindlimbs were paralyzed, the rat could still reach food and water. All experiments were triple blinded: 2 blinded observers gave scores and the third one administered MnTBAP or vehicle, blinded to the 2 observers.

#### BBB test

The motor function of rats was assessed using the BBB locomotor rating scale as reported [56,57]. The test is designed to assess progressive locomotor recovery after SCI. The BBB score was assessed by observing each animal walking in an open field for 4 min. The open field was simply a molded plastic pool with a dimpled floor (100 cm diameter, 21 cm height). To observe animal locomotion requires pretest training. Before surgery, the rats were exposed to the testing environment twice a day for 5 days. Each rat was handled several times during each 30–60 min session so that they no longer showed signs of fear and became accustomed to being picked up and walking continuously in the open field environment. Then the rats were individually placed in the open field for 4 min to assure that all consistently obtained a maximum score of 21. After SCI, the animals were evaluated for hind limb motor function either alone for 4 min or in pairs for 5 min every day for 3 days, then on day 5 and weekly from 1 – 9 weeks by two blinded observers. BBB scores (0 – 21 points) categorize combinations of rat hindlimb movement, trunk position and stability, stepping, coordination, paw placement, toe clearance and tail position, representing sequential recovery stages of the rat’s hind limb motor function after SCI. A score of 0 indicates a paralyzed rat. A score of 21 indicates normal locomotion. Scores from 0 to 8 focus on limb movement of three joints (hip, knee and ankle), representing the early phase of recovery. Scores from 9 to 14 describe the intermediate recovery phase with return of standing and paw placement, stepping with varying degrees of forelimb-hindlimb coordination. Scores from 14 to 20 indicate the rate with return of toe clearance during the step phase, predominant paw position, trunk stability and tail position, reflecting the late and final phase of recovery. Two examiners were positioned across from each other to observe both sides of the rat.

#### Inclined plane test

The inclined plane test measures the animal’s ability to maintain its body position on an inclined board. Each rat was placed on an inclined plane covered by a rubber mat (with 0.6 mm deep grooves) constructed as reported [58,59]. The plane can be adjusted to different slopes to measure the animal’s ability to maintain its body position for 5 sec without falling. The animal was placed on an inclined plane with the head to the right and the left. The average score of the two positions was used. The angle of the board was increased from 0° until it reached an angle at which the rat could maintain its position only for 5 seconds. Final readings to the nearest 5° were taken for stationary positions. All animals were assessed every week for 9 weeks after SCI.

#### The beam walk test

The beam walk was performed as described [60] using seven wooden beams of different widths (1.7, 2.7, 3.7, 4.7, 5.7, 6.7 and 7.7 cm). Before surgery, the rats were trained for 5 days on the plank from the widest to the narrowest for several times each day, so that all of the rats could walk across the 1.7 cm plank. The beam walk performance test was conducted once a week for 9 weeks post-SCI. The animals were allowed to walk on the planks and the narrowest plank on which they could walk without foot slip in two trials was recorded. Rats that could walk only on the widest plank (7.7 cm) were scored 1, and those who could walk on the narrowest plank (1.7 cm) were scored 7.

### Statistical analysis

To obtain the temporal and spatial profiles of cell loss, the counts at each distance were compared with those obtained in the sections at the same distance in the sham control group over time and the counts at each time were also compared over distance by using one-way repeated measures analysis of variance (RMANOVA) followed by the post-hoc Tukey test. Two-way RMANOVA followed by all pairwise multiple comparison using the Bonferroni correction were used to evaluate the spatial protection against different types of cell loss by MnTBAP + L-NA and by MnTBAP alone against apoptosis, and to assess the effect of MnTBAP treatment on the hind limb motor function after SCI by behavioral tests. This statistical procedure was also used to compare DNA fragmentation between the SCI and sham groups at different distances from the epicenter obtained by *ELISA.* Caspase-3 activation in sham controls and by distance from the epicenter was analyzed by analysis of variance (ANOVA) followed by Dunnett’s test. The protection of MnTBAP + L-NA against apoptotic cell death in injured and treated groups was compared by the unpaired t-test. P < 0.05 was considered a statistically significant difference.

## Results

### The quantitative temporal and spatial profiles of motoneuron and glia loss after SCI

To obtain quantitative temporal and spatial profiles of motoneuron and glia cell loss, the spinal cord in injured animals were removed at 1, 6, 12, 24, 48, 72 h and 1 week post-SCI (50 g.cm). The cords in sham-operated controls were removed at 12 h post-laminectomy and served as the control for all time points. The sham control animals underwent the same surgical procedures without weight drop injury. The spinal cords were processed for CV-staining. After staining, the number of cells in the CV-stained sections was counted over time from 1 h to 1 week post-SCI and over distance from 0 to 4 mm caudal from the injury epicenter at 0.5 mm intervals as described in the Methods. The counts were then compared between sham controls and the post-SCI groups for all time points at each distance and by distance at each time point using one way RMANOVA followed by the post-hoc Tukey test.

**Figure 1:**
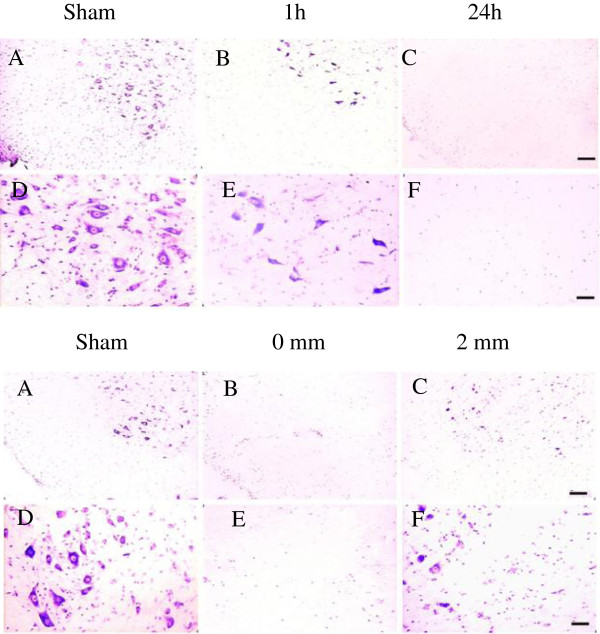
**Photomicrographs of CV-stained spinal cord sections at different times post-SCI and different distances from the epicenter.** Following a laminectomy, the rat spinal cords were injured (50 g.cm), the injured cords were removed at different times post-SCI and processed for CV staining. The upper panel shows surviving cells in CV-stained sections at 1 mm caudal from the epicenter removed at 1 and 24 h post-SCI. The lower panel shows surviving cells in CV-stained sections at 0 and 2 mm caudal from the epicenter removed at 24 h post-SCI. **A-C**: Lower magnification, **D-F**: higher magnification of **A-C**. Scale bar: **A-C**, 100 μm, **D-F**, 50 μm.

Figure 1 illustrates photomicrographs of CV-stained sections at different times post-SCI (upper panel) and at different distances from the epicenter (lower panel). The sham-operated section (A, D) had many cells, including large motoneurons in the ventral gray matter which showed bright nuclei with nucleoli and enrichment in the Nissl bodies. In the upper panel, at 1 h post-SCI, many cells had been lost in the gray matter (B, E) and some neurons lost their normal morphological features. At 24 h post-SCI (C, F), almost no neurons could be observed in the ventral gray matter. In the lower panel, at the epicenter (0 mm), few cells and almost no neurons were observed (B, E). At 2 mm from the epicenter (C, F), some surviving cells including neurons were observed in the ventral gray matter.

**Figure 2:**
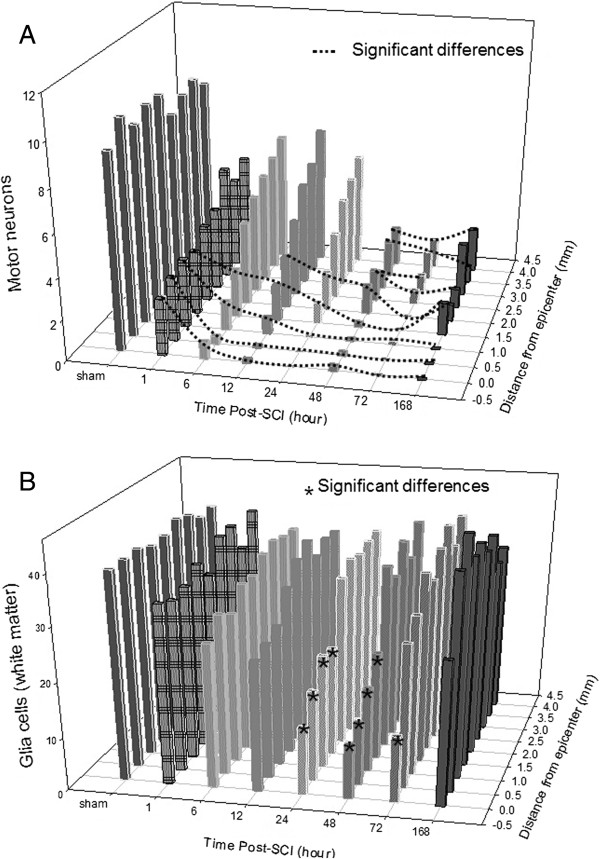
**The temporal and spatial profiles of cell death after SCI.** Cells in the CV-stained sections caudal from the epicenter as shown in Figure 1 in injured or sham control sections were counted as described in the Methods and counts were then statistically compared over time and by distance from the epicenter. The counts were presented as mean ± SD. **A**: The temporal and spatial profile of motoneuron loss in the ventral gray matter of the cord after SCI. The number of surviving motoneurons increased with the distance from the epicenter at each time point, and decreased over time at each distance after SCI. All significant differences were indicated under the dotted lines. **B**: The temporal and spatial profiles of glial cell loss in the ventromedial white matter following SCI. Significant glial loss in the white matter was found only near the epicenter; such loss was not significant at 2 mm from the epicenter at any time post-SCI. * indicates significant difference between sham control and SCI (p < 0.05).

Figure 2 shows the temporal and spatial profiles of cell loss. Figure 2A shows the motoneuron loss in the ventral gray matter following SCI. Neurons larger than 25 μm in diameter in the CV-stained sections were counted as motoneurons. The average counts presented as mean ± standard deviation (SD) of motoneurons at each time and each distance from the epicenter are presented in Table 1. The counts were compared between sham control sections and all post-SCI sections over time at 1, 6, 12, 24, 48 and 72 h, and at 1 week post-SCI for each distance (n = 3 for all). The count at each time point was also compared to that in the sham controls at the same distance. We found significant differences over time at all distances examined (p = 0.02 - <0.001). Motoneurons were significantly fewer at 0 to 1.5 mm from 1 h to 1 week (p = 0.003 - < 0.001); at 2 mm from 12 h to 1 week (p = 0.002 - 0.01); and at 2.5 - 4.0 mm from 48 h to 1 week post-SCI (p = 0.002 - 0.046). Comparisons by distance (0 - 4 mm) from the epicenter at each time point revealed no significant differences in the sham control (p = 0.9) or in the post-SCI groups at 1 h (p = 0.7), 48 h (p = 0.2), 72 h (p = 0.1) and 1 week (p = 0.9).

**Table 1:** The average number of motoneurons in the ventral gray matter of rat spinal cords

**Time post-SCI (h)**	**Distances caudal from the epicenter (mm)**								
	**0.0**	**0.5**	**1.0**	**1.5**	**2.0**	**2.5**	**3.0**	**3.5**	**4.0**
Sham	9.3 ± 1.0	10.2 ± 1.3	9.4 ± 0.3	9.8 ± 0.8	9.8 ± .8	8.4 ± 1.5	8.9 ± 1.0	9.2 ± 0.7	8.6 ± 0.3
1	2.6 ± 2.2	3.1 ± 2.0	3.3 ± 0.3	3.1 ± 2.1	3.7 ± 2.0	3.9 ± 1.3	5.4 ± 1.3	4.3 ± 1.3	4.9 ± 0.4
6	0.8 ± 1.3	0.4 ± 0.7	1.4 ± 1.3	2.3 ± 2.0	4.0 ± 1.2	4.7 ± 0.8	5.2 ± 1.6	5.6 ± 2.1	6.1 ± 3.5
12	0.2 ± 0.2	0.1 ± 0.3	0.8 ± 1.2	1.7 ± 1.2	2.5 ± 2.7	3.7 ± 2.8	4.9 ± 2.8	5.4 ± 2.4	6.6 ± 1.6
24	0	0	0	1.04 ± 1.9	1.9 ± 2.5	3.2 ± 2.5	4.3 ± 2.2	4.8 ± 2.6	5.4 ± 1.0
48	0.3 ± 0.3	0.2 ± 0.3	0.1 ± 0.1	0.22 ± 0.3	1.4 ± 1.2	1.5 ± 1.2	0.8 ± 0.6	1.7 ± 1.1	1.9 ± 0.8
72	0	0	0.2 ± 0.4	0.1 ± 0.1	0	0.7 ± 0.9	0.8 ± 1.1	1.4 ± 0.3	1.4 ± 1.1
168	0	0	0	1.6 ± 2.7	0.8 ± 1.3	0.9 ± 3.1	2.5 ± 3.7	0.8 ± 0.6	2.1 ± 0.8

The number of motoneurons at different times post-SCI and different distances from the epicenter was presented as mean ± SD (n = 3 for all).

The absence of significant differences over distance and the small number of surviving motoneurons at times from 48 h to 1 week show that most motoneurons had been lost by 48 h to at least 4.0 mm from the epicenter. Significant differences were found at 6, 12 and 24 h (p = 0.03 - <0.001) post-SCI. At 6 h, the difference was significant over distance (p = 0.03), but no difference was found among sections; at 12 h, the difference was significant over distance (p < 0.001) with significantly more motoneurons at 3 mm than at 0 and 0.5 mm (p = 0.03 for both), at 3.5 mm than at 0.5 - 1 mm (p = 0.01 - 0.04) and at 4 mm than at 0–1.5 mm (p = 0.001- 0.03); at 24 h post-SCI, a significant different was found only between 4 mm and 0–1 mm. By 48 h to 1 week, no significant differences were found over distance (p = 0.1 - 0.9), indicating that most motoneurons had already been lost at all distance examined.

Figure 2B shows the temporal and spatial profile of glial cell loss in the ventral white matter following SCI. Glial cells in the ventral white matter of CV-stained sections were counted as described in the Methods. The average counts (mean ± SD) at each time and each distance from the epicenter are presented in Table 2. Significant glial cell loss appeared at 24–72 h post-SCI in 0 mm sections (p = 0.03 - 0.01) and at 24 and 48 h post-SCI in 0.5 - 1.5 mm sections compared to sections from sham-operated controls (p = 0.047 - 0.003). At 72 h post-SCI, glial cells began proliferating and significantly more glial cells were found at 72 h than at 24 and 48 h post-SCI, but only in 1.5 mm sections (p = 0.02-0.03). By 1 week, the number of glial cells at 0.5 mm was significantly higher than at 24 and 48 h (p = 0.006 - 0.03); higher at 1 mm than at 6–48 h; and higher at 1.5 mm than at 24 and 48 h (p = 0.047 - <0.001) post-SCI. Glial loss in the ventral white matter was much slighter than motoneuron loss in the ventral gray matter, and the loss was quickly offset by proliferation (n = 3 for all time points and all distances).

**Table 2:** The average number of glial cells in the ventral white matter of rat spinal cords

**Time post-SCI (h)**	**Distances caudal from the epicenter (mm)**								
	**0.0**	**0.5**	**1.0**	**1.5**	**2.0**	**2.5**	**3.0**	**3.5**	**4.0**
**Sham**	41.4 ± 0.7	41.3 ± 1.8	41.6 ± 2.5	40.0 ± 1.3	40.5 ± 0.9	42.3 ± 1.5	41.1 ± 2.7	39.3 ± 2.4	40.0 ± 3.1
**1**	35.7 ± 4.9	34.2 ± 0.4	34.8 ± 3.1	37.2 ± 1.2	33.2 ± 0.7	37.9 ± 0.4	39.4 ± 0.4	39.3 ± 0.5	39.2 ± 1.6
**6**	28.9 ± 4.0	32.0 ± 3.8	30.1 ± 8.3	33.8 ± 4.2	33.5 ± 2.2	34.6 ± 3.2	36.7 ± 0.7	34.8 ± 2.4	35.6 ± 0.3
**12**	26.1 ± 8.1	27.0 ± 5.4	28.3 ± 6.7	33.5 ± 4.0	37.2 ± 4.9	34.1 ± 3.1	34.2 ± 3.3	35.7 ± 2.8	35.7 ± 0.3
**24**	13.7 ± 6.6	18.4 ± 1.1	23.0 ± 3.9	22.2 ± 4.2	34.1 ± 5.5	36.8 ± 4.8	35.1 ± 2.2	35.7 ± 4.0	36.2 ± 0.3
**48**	10.9 ± 5.3	12.7 ± 6.1	17.2 ± 8.0	21.8 ± 9.7	36.8 ± 3.4	34.4 ± 5.3	38.1 ± 0.8	32.3 ± 0.7	38.5 ± 0.8
**72**	14.0 ± 1.5	28.3 ± 11.5	32.1 ± 9.0	38.1 ± 6.2	34.8 ± 4.5	39.3 ± 4.3	41.7 ± 2.07	37.3 ± 0.7	40.3 ± 4.4
**168**	28.7 ± 21	42.8 ± 17.3	47.6 ± 3.4	41.7 ± 5.4	42.2 ± 7.0	39.4 ± 4.3	40.2 ± 2.8	34 ± 8.1	34 ± 7.2

The number of glial cells at different times post-SCI and different distances from the epicenter was presented as mean ± SD (n = 3 for all).

### Morphological characterization of SCI-induced neuronal and glial apoptosis

By counting TUNEL-positive cells in the sections 0, 1, 2 and 4 mm from the epicenter in the cord removed at 1 h to 1 week post-SCI, we found that the number of TUNEL-positive cells in the ventral gray matter peaked at 12 to 48 h post-SCI and maximized at the distances of 1 and 2 mm from the epicenter [43]. Therefore, in this study, neuronal and glial apoptosis was further characterized morphologically as described in the Methods at 12 or 24 h post-SCI and at 2 mm rostral from the epicenter by the following staining: 1) TUNEL and immunohistochemical double staining with antibodies against specific cell markers of different types of cells (anti-NSE for neurons, anti-ChAT for motoneurons and anti-GFAP for astrocytes); 2) TUNEL and anti-ChAT immuno-histochemical and immuno-fluorescence double staining of apoptotic motoneurons; and 3) active caspase-3 and specific cell marker double immuno-fluorescence staining of apoptotic neurons and astrocytes. Neuronal and glial apoptosis was confirmed by transmission electron microscopy.

**Figure 3:**
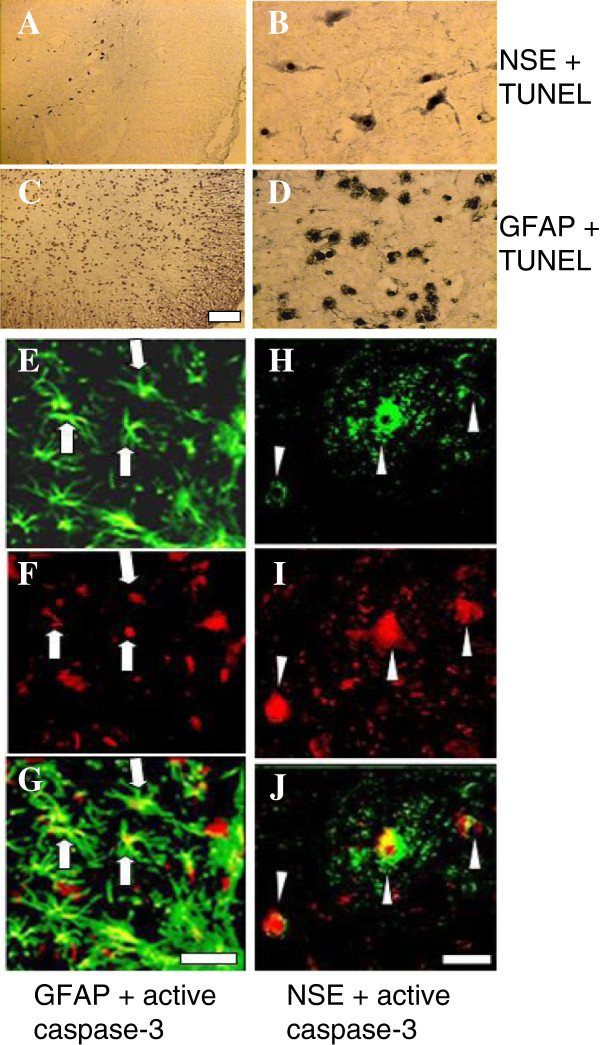
**Photomicrographs of the morphological characterization of apoptotic neurons and astrocytes. A-D**: photomicrographs of TUNEL and immunohistochemical double-stained sections 2 mm rostral from the epicenter in the ventral horn of injured spinal cords removed at 24 h post-SCI. **A** and **B**: TUNEL and NSE double-stained TUNEL-positive neurons at lower **(A)** and higher **(B)** magnifications. The arrowheads indicate TUNEL-positive nuclei in NSE-positive neurons. **C** and **D**: TUNEL and GFAP double-stained TUNEL-positive astrocyte at lower **(C)** and higher **(D)** magnifications. The arrows indicate TUNEL-positive nuclei in GFAP-positive astrocytes. **E-J**: photomicrographs of active caspase-3 and immuno-fluorescence double-stained sections 2.05 mm rostral from the epicenter in the ventral gray matter of injured spinal cords removed at 12 h post-SCI. **E-G**: p20 fragment and GFAP double-stained active caspase-3 with active astrocytes. **E**: active astrocytes (GFAP-positive, arrows); **F**: the same astrocytes stained for p20 (arrows). **G**: immuno-colocalization of **E** and **F**, showing caspase-3-positive astrocytes (arrows, yellow). **H-J**: p20 fragment and NSE double-stained neurons. **H**: NSE-positive neurons (arrowhead); **I**: the same neurons were also p20-positive (arrowhead). **J**: immuno-colocalization of **H** and **I** showing caspase-3-positive neurons (arrowhead, yellow). Scale bar = 100 μm.

Figure 3 illustrates the photomicrographs of the morphological characterization of apoptosis in neurons and astrocytes. DNA fragmentation is an event of apoptosis and TUNEL stains the fragmented DNA in the nuclei. Anti-NSE and anti-GFAP immunohistochemical staining identifies neurons and reactive astrocytes, respectively. A to D in Figure 3 show the photomicrographs of TUNEL and NSE or GFAP immunohistochemically double-stained sections in the ventral horn of the gray matter in an injured spinal cord sections 2 mm rostral from the epicenter removed at 24 h post-SCI. The arrowheads in B indicate TUNEL-positive nuclei in NSE-positive neurons. The arrows in D indicate TUNEL-positive nuclei in GFAP-positive astrocytes. These double-stained spinal cord sections localize the apoptotic DNA fragmentation in the nuclei of neurons (A and B) and astrocytes (C and D). This is the morphological indication of neuronal and glial apoptosis. Since caspase-3 activation occurs prior to DNA fragmentation, the injured spinal cord was removed at 12 h post-SCI in this study for active caspase-3 staining. E to J in Figure 3 show the photomicrographs of active caspase-3 and NSE or GFAP immuno-fluorescence double-stained apoptotic astrocytes (E - G) and neurons (H - J), respectively, in the spinal cord sections 2.05 mm rostral from the epicenter removed at 12 h post-SCI. E to G show double immuno-fluorescence-stained sections with antibodies against an active fragment p20 of caspase-3 and anti-GFAP. E shows astrocytes (GFAP-positive, arrows) and F shows the same astrocytes stained for p20 (arrows). G is the colocalization of E and F, showing caspase-3-positive astrocytes (arrows). H to J show double immuno-fluorescence-stained neurons. H shows NSE-positive neurons (arrow head). I shows that the same neurons are also p20-positive (arrow head). J is colocalization of H and I, showing active caspase-3-positive neurons (arrow head). These double immuno-fluorescence staining co-localized caspase-3 activation in the neurons and astrocytes and provided another morphological indication of neuronal and glial apoptosis.

**Figure 4:**
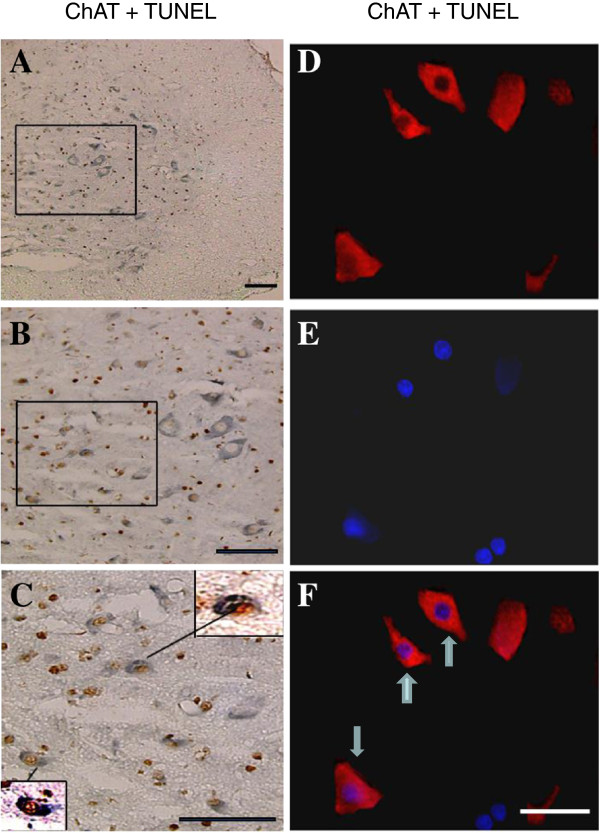
**Photomicrographs of the morphological characterization of apoptotic motoneurons. A-C**: Photomicrographs of TUNEL and ChAT double immunohistochemical stained section at 2 mm rostral from the epicenter removed at 24 h post-SCI. **A**: lower magnification of the ventral gray matter; **B**: higher magnification corresponding to the frame in **A**; **C**: high magnification corresponding to the frame in **B**. Two enlarged TUNEL-positive motoneurons were shown in the frames of **C**. Scale bar, 100 μm. **D-F**: Photomicrographs of TUNEL and ChAT double immuno-fluorescence stained section at 2.05 mm rostral to the epicenter removed at 24 h post-SCI. The section was double immuno-stained with anti-ChAT primary antibody (**D**, red) and TUNEL (**E**, blue), followed by anti-mouse Alexa Fluor 594 secondary antibody. **F**: immuno-colocalization of **D** and **E** showing apoptotic motoneurons (bluish red) in the ventral gray matter of the cord from a vehicle control rat. Photomicrographs are taken at high magnification (scale 100 μm).

In the spinal cord, most ChAT immuno-reactive neurons are motoneurons. Using ChAT and TUNEL double-staining, we found that a number of ChAT-positive neurons were also TUNEL-positive in the ventral horn of spinal cord sections 2 mm rostral from the epicenter removed at 24 h post-SCI, indicating motoneuron apoptosis (Figure 4A-C). Motoneuron apoptosis was further shown by the ChAT and TUNEL double immuno-fluorescence colocalization (Figure 4D-F). ChAT-immuno-staining showed red cell bodies of motoneurons (D) and TUNEL staining showed blue nuclei (E) in the ventral horn of the gray matter of the cord from a section 2.05 mm rostral from the epicenter removed at 24 h post-SCI. The blue TUNEL-stained nuclei were localized clearly in the red ChAT-positive cell bodies of motoneurons (F. arrows).

**Figure 5:**
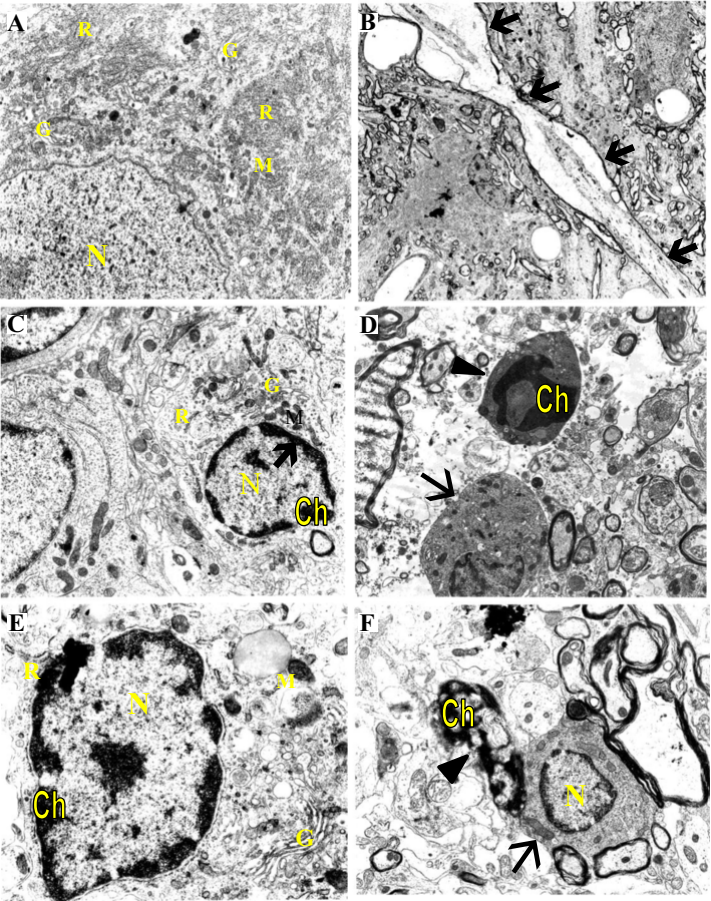
**TEM confirmation of neuronal and glial apoptosis.** The tissue for TEM was obtained at 24 h post-SCI from layer VIII of the ventral horn of the cord and the 2–2.5 mm rostral from the epicenter were processed for TEM as described in the Methods. **A**: a normal neuron from sham control (×14,300). **B**: a degenerating neuronal axon (×3,300). **C** (×24,475); **D** (×14,300); **E** (×42,625); **F** (×18,150): different stages of apoptotic neurons and glial cells. Symbols: N-Nucleus, M-Mitochondria, R- Rough endoplasmic reticulum, G-Golgi complex, Ch-Chromatin.

SCI-induced apoptosis was confirmed by TEM identification based on the specific morphology of apoptosis. Figure 5 illustrates the ultra-fine structural changes in apoptotic cells in layer VIII of the ventral horn in the gray matter of the spinal cord sections at 2-2.5 mm rostral from the epicenter removed at 24 h post-SCI (50 g.cm). Figure 5A, a sham control section 2 mm rostral from the epicenter, shows a normal neuron with a large, euchromatic (pale-staining) nucleus (N), abundant rough endoplasmic reticulum (R), mitochondria (M), and Golgi complexes (G) in the cytoplasm. Figure 5B-F show different stages of apoptosis and degeneration from injured animals. B shows a degenerating swollen axon (arrow) with vacuoles. C-F shows very early to late stages of apoptotic degeneration of neuronal and glial cells. C shows a very early stage apoptotic neuron with condensed chromatin (Ch) in the rim of the nucleus (arrow) and unchanged organelle in the cytoplasm. D shows a normal glial cell (arrow) with a smaller cell body and heterochromatic nucleus stained more intensely than the nucleus of the neuron. An apoptotic glial cell near the normal glial cell (arrow head) is shrinking, becoming denser and the chromatin undergoing condensation to form an irregular dense mass while the organelles are becoming indiscernible. E shows the early stage of an apoptotic neuron with condensed chromatin accumulated mainly at the nuclear rim; the organelles are intact. F represents a nucleus which is fragmented and has formed highly condensed apoptotic bodies (arrow head). A phagocyte (arrow) is shown engulfing the apoptotic body.

### Biochemical characterization of apoptosis in spinal cord tissues following SCI

Apoptotic DNA fragmentation was characterized by specific laddering of radiolabeled DNA fragments and quantitated by *ELISA*. Activation of caspase-3 was also analyzed biochemically. As described in the Methods, after impact injury to the rat spinal cords, the cords were frozen *in situ* and cut into three sections of equal length (1 cm) with section 1 centered at the injury epicenter and sections 2 and 3 adjacent and caudal to section 1. Since the number of TUNEL-positive cells peaked at 12 – 48 h post-SCI [43], in this study, DNA fragmentation was characterized at 48 h post-SCI or post-laminectomy for sham controls. Since apoptosis appeared as early as 4–6 h post-SCI [4,43] and caspase activation occurs prior to apoptotic DNA fragmentation, caspase-3 activity was analyzed at 4 h post-SCI.

**Figure 6:**
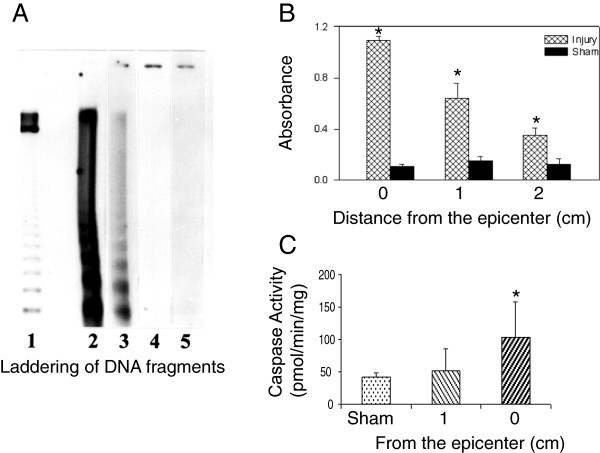
**Biochemical evidence of apoptosis.** The spinal cord was removed at 48 h post-SCI by being frozen in situ with liquid nitrogen and divided into three sections of equal length (1 cm each) with section 1 centered at the epicenter and sections 2 and 3 centered at 1 and 2 cm caudal to the epicenter for DNA laddering and *ELISA* as described in the Methods. **A**: The laddering of DNA fragments in the spinal cord tissue. Lane 1, standard DNA marker; lane 2, the tissue obtained from the epicenter; lanes 3 and 4, tissue centered 1 and 2 cm away from the epicenter, respectively; and lane 5, tissue from a sham-operated rat. The fragmented DNA shown in each lane was obtained from the corresponding sections of one rat (60 mg wet weight tissue). The results are representative of four separate experiments. **B**: Quantitative analysis of fragmented DNA by *ELISA* with 3 sections at epicenter, 1 and 2 cm caudal from the epicenter. Each sample for *ELISA* analysis equaled 2 mg wet weight tissue. Error bars: mean ± SEM. Both A and B show significantly decreased DNA fragmentation with an increase in distance from the epicenter. **C**: Caspase-3 activation analyzed at 4 h post-SCI. The injured cord was divided into two sections with section 1 centered at the injury epicenter and section 2 centered 1 cm from the epicenter. Error bars: mean ± SEM. The caspase-3-like protease activity was significantly higher at the injury epicenter compared to 1 cm from the epicenter and sham control.

Figure 6 represents the results of our biochemical analysis. Figure 6A shows autoradiographs of DNA fragments analyzed by agarose gel electrophoresis from the cords extracted at 48 h post-SCI (lanes 2 – 4) and the sham-operated control (lane 5). Fragmented DNA displayed a ladder-like electrophoretic pattern. The intensity of the ladder was higher in lane 2 (epicenter) than in lane 3 (section 2 – 1 cm from the epicenter). No fragmented DNA bands were observed in lane 4 (section 3 – 2 cm from the epicenter). The sham-operated sample (lane 5) did not show any detectable fragmentation. DNA fragmentation was quantitated by *ELISA* (Figure 6B). The cellular DNA isolated from three sections of injured spinal cord tissue showed a significantly higher absorbance at 405 nm than did the three corresponding sections in the sham-operated rats (n = 6, p < 0.001, RMANOVA followed by all pairwise multiple comparison with the Bonferroni correction). The average increases in the three injured sections were 2- to 10-fold that of corresponding sham controls. Fragmented DNA in the injured tissue was more abundant in the epicenter (section 1) than in 1 (section 2) and 2 (section 3) cm from the epicenter (p < 0.001), and in 1 cm than in 2 cm caudal from the epicenter (p = 0.01). The decline over distance from the injury site measured by *ELISA* (Figure 6B) was comparable to the decline in intensity of DNA laddering over the same distance (Figure 6A). Figure 6C shows caspase-3 activation at 4 h post-SCI. The caspase-3-like protease activity was measured as described in the Methods. The caspase-3-like protease activity was significantly higher in the epicenter (section 1) of the cords than at 1 cm from the epicenter (section 2) in the injured cords (n = 5) or in the sham control (n = 4, p = 0.03 for both). Results for the sham control and 1 cm from the epicenter did not differ significantly.

### MnTBAP + L-NA treatment reduced secondary cell loss and the number of TUNEL-positive cells after SCI

**Figure 7:**
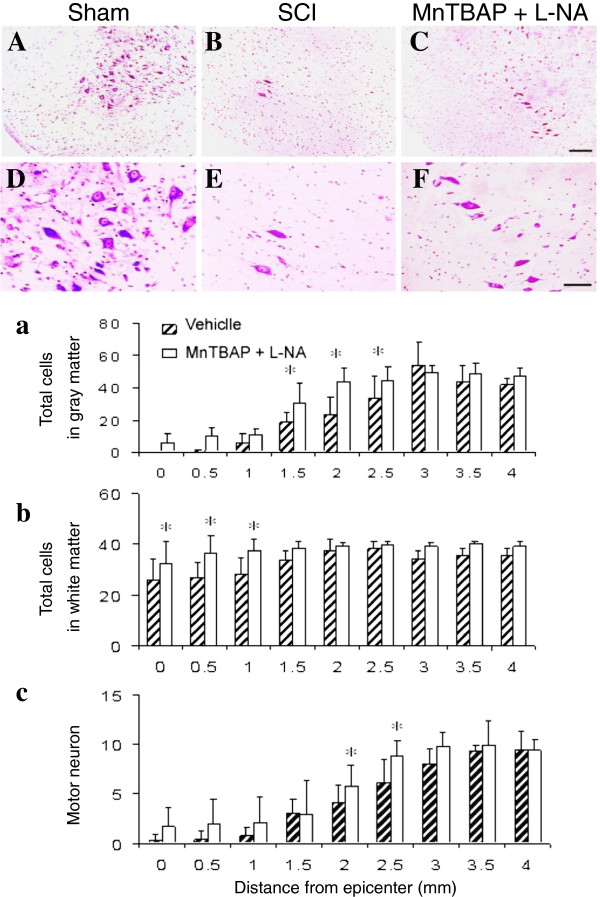
**Reduction of SCI-induced cell loss by MnTBAP + L-NA.** MnTBAP (10 mg/kg) plus L-NA (1 mg/kg) was given i.p. at 15 min pre- and 6 h post-SCI (50 g.cm) and the rats were sacrificed at 12 h post-SCI. Upper panel: photomicrographs of the ventral gray matter in the CV-stained spinal cord sections at 2 mm rostral from the epicenter. **A-C**: lower magnification, **D-F**: higher magnification of A-C. Clearly, the combination of MnTBAP and L-NA treatment increased the number of surviving cells in the gray matter of the cord. Scale bars shown in C and F: 100 and 50 μm, respectively. Lower panel: spatial protection by MnTBAP + L-NA against losses of total cells in gray matter **(a)**, glial cells in white matter **(b)**, and motoneurons **(c)**. * indicates a significant difference between the combination-treated and the vehicle-treated groups (p < 0.05). Error bars, mean ± SEM. Compared to vehicle-treated rats, the combination significantly reduced cell loss in both the gray and the white matter.

For the reason mentioned in the Background, a combination of MnTBAP (10 mg/kg) plus L-NA (1 mg/kg) was first tested for its ability to protect against cell loss. The combination was given (i.p) at 15 min pre- and 6 h post-SCI (50 g.cm), then the cord was removed at 12 h post-SCI for CV-staining. The total surviving cells and motoneurons (neurons larger than 25 μm in diameter) were counted in the gray matter, and the surviving glial cells were counted in the white matter in the CV-stained sections 0 – 4 mm rostral from the epicenter at 0.5 mm intervals. The upper panel of Figure 7 provides photomicrographs of CV-stained sections showing that MnTBAP + L-NA treatment reduced cell loss in sections 2 mm rostral from the epicenter in the ventral gray matter of the cord at 12 h post-SCI. Many motoneurons were observed in the ventral gray matter of the cord in the sham control sections (A, D). Dramatic motoneuron loss was observed in the vehicle-treated group (B, E). The motoneuron loss was attenuated by the MnTBAP + L-NA treatment (C, F), suggesting that RS contribute to motoneuron loss in SCI. The number of cells in sections at different distances from the epicenter were compared between the combination-treated (n = 6) and the vehicle-treated (n = 5) groups using two-way RMANOVA followed by pairwise multiple comparisons with the Bonferroni correction. We found that the combination treatment significantly reduced: total cell loss in the gray matter of sections 1.5 – 2.5 mm rostral from the epicenter (p = 0.02 - < 0.001, Figure 7a); glial cell loss in the ventromedial white matter of sections 0 - 1.0 mm rostral from the epicenter (p = 0.01 - 0.002, Figure 7b); and motoneuron loss at 2.0 and 2.5 mm from the epicenter (p < 0.05 and p = 0.01, Figure 7c) compared to vehicle-treated rats (lower panel, Figure 7).

**Figure 8:**
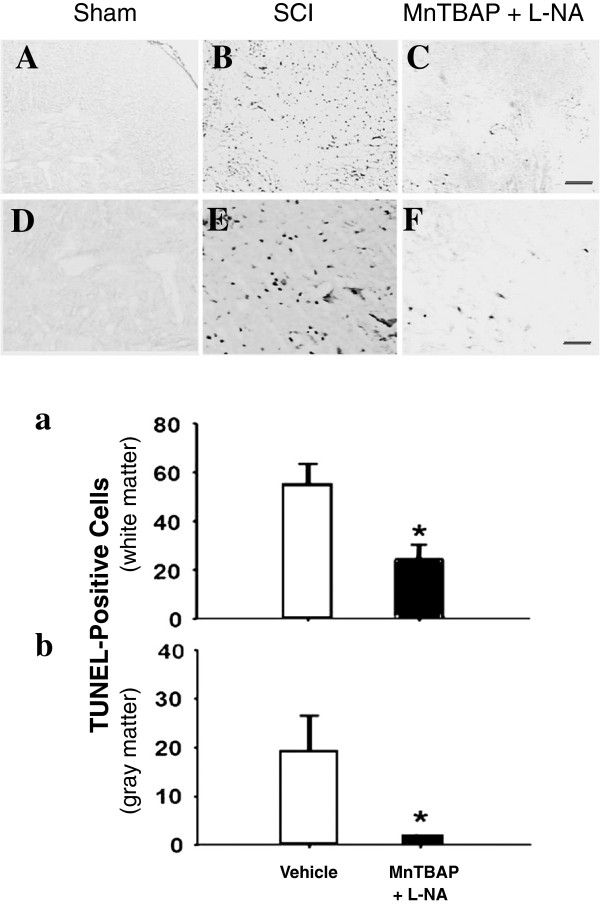
**MnTBAP + L-NA reduced SCI-induced apoptosis.** Spinal cord sections were obtained at 12 h post SCI as in Figure 7. Upper: photomicrographs of TUNEL-stained sections at 2 mm rostral from the epicenter in the gray matter of the cord. **A-C**: lower magnification and **D-F**: higher magnification of A-C. **A** and **D**: sham control; **B** and **E**: injured section; **C** and **F**: injured section treated with MnTBAP + L-NA. Treatment by MnTBAP + L-NA clearly reduced the number of TUNEL-positive cells. The TUNEL-positive cells at the sections 2 mm from the epicenter were counted in both the gray and the white matter and the counts compared between the combination-treated and vehicle-treated groups (lower panel). **a**: Number of TUNEL-positive cells in the gray matter. **b**: Number of TUNEL-positive cells in the white matter. Error bars, mean ± SEM. Asterisk indicates a significant difference between the two treatment groups (p < 0.05). The combination of MnTBAP + L-NA significantly reduced the number of TUNEL-positive cells in both the gray and the white matter.

Since the number of TUNEL-positive cells in the gray matter peaked from 12 to 48 h post-SCI [43], the effect of MnTBAP + L-NA on apoptosis was evaluated at 12 h post-SCI by counting the TUNEL-positive cells in the gray and white matter in the vehicle- and combination-treated sections as described above (Figure 8). The upper panel of Figure 8 provides photomicrographs of TUNEL-stained sections 2 mm rostral from the epicenter in the gray matter of the cord at 12 h post-SCI. The sham control sections showed no TUNEL-positive cells in the gray matter of the cord (A, D). In the SCI group (B, E), many TUNEL-positive cells appeared. Treatment with the combination clearly reduced the number of TUNEL-positive cells (C, F). The cells in the sections 2 mm rostral from the epicenter in the vehicle-treated (n = 5) and the combination-treated groups (n = 6) were counted and the counts compared using unpaired *t*-test. The quantitative comparison demonstrated that the MnTBAP + L-NA treatment significantly (p = 0.01) reduced the number of TUNEL-positive cells in the gray (Figure 8a) and white (Figure 8b) matter, suggesting that RS contribute to apoptotic DNA fragmentation following SCI.

### MnTBAP alone reduced the number of TUNEL-positive cells after SCI

**Figure 9:**
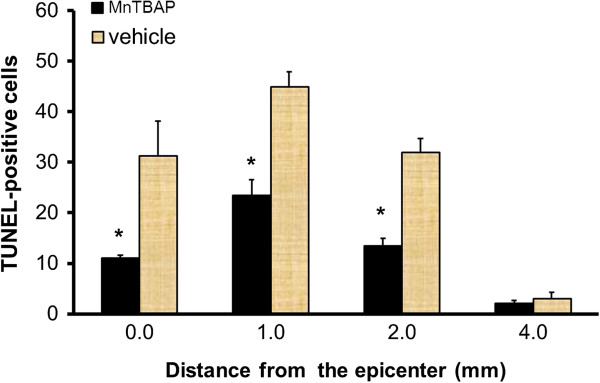
**MnTBAP protection against apoptotic cell loss following SCI.** MnTBAP (10 mg/kg, ip) was administered as in Figures 7 and 8 and the rats were sacrificed at 24 h post-SCI. The number of TUNEL-positive cells was counted in the gray matter in the sections at 0 – 4 mm rostral from the epicenter at 1 mm intervals. The counts in MnTBAP-treated sections were compared with those in vehicle-treated sections. * indicates a significant difference between the MnTBAP-treated and the vehicle-treated groups (P < 0.05). Error bars, mean ± SEM. Compared to vehicle-treated rats, 10 mg/kg MnTBAP significantly reduced the number of TUNEL-positive cells.

To test the effect of MnTBAP treatment on cellular apoptosis, the cords of MnTBAP-treated and vehicle-control animals (n = 5 for both groups) were removed at 24 h post-SCI. The sections at 0–4 mm rostral from the epicenter in the two groups of animals were stained with TUNEL and the number of TUNEL-positive cells was counted (Figure 9). The counts in MnTBAP-treated sections were compared with those in vehicle-treated sections by two-way RMANOVA followed by pairwise multiple comparisons with the Bonferroni correction. We found that 10 mg/kg MnTBAP given i.p. significantly (p = 0.01) reduced the number of TUNEL-positive cells over distance compared with the vehicle-treated group. Comparisons by treatment (saline vs. MnTBAP) using pairwise multiple comparisons indicated a significant difference (p < 0.001). Comparisons by distance within the saline group indicated that significantly more TUNEL-positive cells appeared at the distances of 0, 1 and 2 mm than at 4 mm from the epicenter (p < 0.001 for all) with no significant difference in number at shorter distances from the epicenter, indicating that injury-induced apoptosis extended from the epicenter to less than 4 mm away. Comparisons by distance within the MnTBAP treatment group indicated that significantly more TUNEL-positive cells appeared at 1 mm only than at 4 mm from the epicenter (p < 0.001), suggesting the protective effect of MnTBAP. Comparisons of the number of TUNEL-positive cells between saline- and MnTBAP-treated sections at each distance rostral from the epicenter demonstrated that MnTBAP significantly reduced the number of TUNEL-positive cells at 0, 1, and 2 mm (p < 0.001, for all), but not at 4 mm, from the epicenter with the counts of 31 ± 12, 45 ± 5, 32 ± 5 and 3 ± 2 at 0, 1, 2 and 4 mm, respectively, rostral from the epicenter in the vehicle-treated group reduced to 11 ± 0.6, 23 ± 3.2, 13 ± 1.6 and 2 ± 0.6 (mean ± SEM) in the corresponding sections in the MnTBAP-treated group. In other words, the counts of TUNEL-positive cells in vehicle-treated sections were approximately 3, 2 and 2.5 times higher at 0, 1 and 2 mm from the epicenter than those in the corresponding sections of the MnTBAP-treated groups. This again indicates that RS contribute to apoptotic DNA fragmentation following SCI.

### MnTBAP improved functional recovery after SCI

SCI-induced neurological deficits and the ability of MnTBAP alone to protect against such dysfunction were evaluated by the BBB test, beam walk test and the inclined plane test for 9 weeks. Behavioral test scores were compared by two-way RMANOVA followed by pairwise multiple comparisons between MnTBAP (n = 8) and sham (n = 4), saline (n = 7) and sham, and MnTBAP and saline groups. Eight rats were used for behavioral tests in the injured groups. However, due to the loss of body weight, one rat in the vehicle-treated group was terminated. Therefore the data obtained in this group were from 7 rats.

#### MnTBAP significantly improved the BBB scores

**Figure 10:**
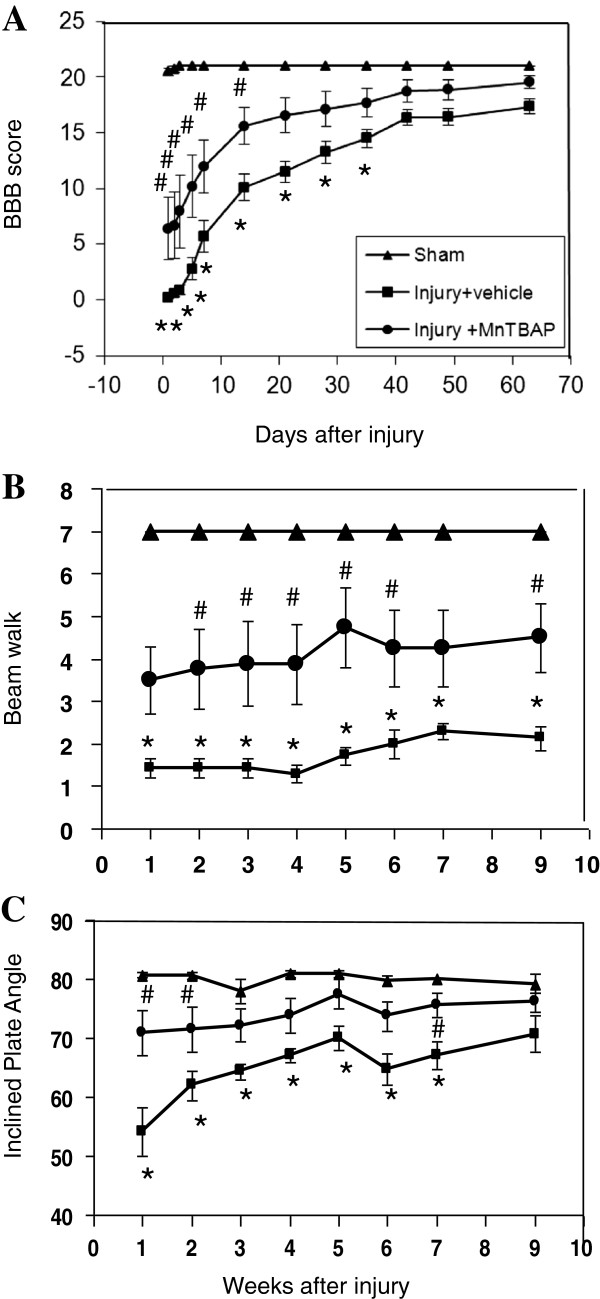
**MnTBAP protection against functional deficits following SCI.** The spinal cords of rats were injured at vertebra T10 (12.5 g.cm) using the NYU device. The behavioral tests were performed on animals treated with MnTBAP and saline, and on sham control animals as described in the Methods. MnTBAP (10 mg/kg) or saline was given by i.p. injection at 15 min pre- and 4 h post-SCI for behavioral tests. All experiments were triple blinded. **A**: results of the BBB test. **B**: results of the beam walk test. **C**: results of the inclined plane test. Clearly, MnTBAP treatment significantly improved the functional recovery following SCI relative to the vehicle-treated animals in all three tests. Error bars: mean ± SEM. * indicates a significant difference between the groups of injured with vehicle-treated and the sham controls. # indicates a significant difference in the injured groups between vehicle-treated and MnTBAP-treated.

The rats were evaluated for hind limb motor function pre-SCI and at 1, 2, 3 and 5 days and 1–9 weeks post-SCI (Figure 10A) as described in the Methods. All animals started with baseline BBB scores of 21. In the injured with vehicle-treated group, all animals manifested bilateral hind limb paralysis immediately following injury and gradually recovered. The overall scores in the sham-operated group were significantly higher than in the vehicle-treated group (p < 0.001) and the MnTBAP-treated group (p = 0.01); the sham-operated group had no significant differences in scores over time during the 9 weeks of observation and no significant difference in scores at any time point, as expected. In the vehicle-treated SCI group, the BBB scores: at 9 weeks were significantly higher than at 1–5 days and 1–4 weeks (p = 0.02 for 4 weeks and p < 0.001 for the rest); at 7 and 6 weeks were significantly higher than at 1–5 days and 1–3 weeks (p = 0.001 for 3 weeks and p < 0.001 for the rest); at 5 weeks were significantly higher than at 1–5 days and 1–2 weeks (p = 0.006 for 2 weeks and p < 0.001 for the rest); and at 2–4 weeks were significantly higher than at 1–5 days and 1 week (p = 0.008 for 1 to 2 weeks and p < 0.001 for all the rest). Even at 1 week, the scores were significantly higher than at 1–3 days (p = 0.001 for 3 days and p < 0.001 for 1 and 2 days).

Scores did not differ significantly over 1 to 5 days. The scores gradually increased from very low during the first 5 days to higher scores by 9 weeks, indicating a gradual recovery even without treatment. As early as the first day post-injury, the MnTBAP-treated rats showed more movement of their hind limbs than did vehicle-treated animals. MnTBAP treatment significantly increased the overall BBB scores compared with the vehicle-treated group over time (p < 0.001) with significantly greater scores at days 1 to 5 (p = 0.003 - 0.01) and at weeks 1 and 2 (p = 0.01 and 0.03). At each time point during functional recovery, the scores in the MnTBAP-treated group were consistently better than those in vehicle-treated animals.

#### MnTBAP significantly increased the beam walk scores

The beam walk performance was tested weekly at 1 to 9 weeks post-SCI (Figure 10B) as described in the Methods. The rats were pre-trained for 5 days so that all of them could walk through the narrowest beam (1.7 cm). The 4 sham-operated rats retained this ability during the entire study. SCI significantly (p < 0.001) decreased the score of the beam walk test in injured animals compared with the sham-operated controls, dropping immediately after injury and continuing at all time points (p < 0.001 for all). MnTBAP treatment significantly increased the overall scores (p = 0.02) reduced by SCI within 9 weeks post-SCI with significant increases at weeks 2 to 6 (p = 0.04 – p = 0.005), but not at weeks 1 (p = 0.07) and 7 (p = 0.09).

#### MnTBAP significantly increased the angles of inclined plane

This test was performed weekly at 1 to 9 weeks post-SCI (Figure 10C). In the sham-operated control group, all animals remained at the maximum angle to stay on the inclined plane at all times. Injury significantly (p < 0.001) reduced the maximum angle from week 1 to 7 compared with sham controls (p = 0.03 - <0.001). MnTBAP treatment significantly increased the angles over time compared to the vehicle-treated groups (p = 0.01), but not in comparison to the sham groups (p = 0.10). MnTBAP treatment significantly increased the angle of the inclined plane at weeks 1–2 and week 7 post-SCI (p = 0.04 - p < 0.001) relative to vehicle-treated animals.

## Discussion

Using a weight drop SCI model, the present study established the temporal and spatial profiles of motoneuron and glial cell loss after SCI, demonstrated that apoptosis is the common mechanism for different types of cell death after SCI, and characterized and quantitated apoptosis at different distances from the injury epicenter. The ability of the catalytic antioxidant MnTBAP and of the combination MnTBAP + L-NA to protect against SCI-induced cell loss and apoptosis were explored. The effectiveness of MnTBAP alone to attenuate neurological dysfunction was also evaluated.

Comparing our temporal and spatial profile of motoneuron loss following SCI in the present study (Figure 2A) with the profile of total neuron loss that we reported previously [43], we found that motoneuron loss had the same pattern as neuron loss: increase over time and decrease over distance from the epicenter. However, the significant loss of motoneurons occurred earlier and spread away from the epicenter quicker than total neuron loss. From 48 h until a week following SCI, motoneuron loss did not significantly differ by distance, indicating that most motoneuron loss occurred within this period. Although motoneurons are located in the ventral gray matter, farther from the impact site than total neurons, motoneuron loss was more severe than total neuron loss (Figure 2A). Our quantitative profile of motoneuron loss extended the information reported hitherto [61] from 24 h to one week.

Compared with the neuron and motoneuron loss in the ventral gray matter, our temporal and spatial profile of glial loss in the ventral white matter (Figure 2B) showed a different pattern: a delayed (starting at 24 h post-SCI), much diminished and more concentrated at the epicenter (at 0 - 1.5 mm from the epicenter) and quickly decreased (by 72 h post-SCI) due to proliferation. One report [61] found significant glia loss in the ventromedial white matter at 15 min after thoracic SCI. Our observation of significant glial loss starting at 24 h post-SCI may be attributed to the differences in the impact site, as Bresnahan et al. [62] indicated that a similar impact force produces shorter, more truncated lesion sites at lumbar levels with less involvement of the white matter than in thoracic lesions, as determined by the three-dimensional structure of lesion sites [62]. The injury site in most previous studies of SCI was at vertebrae T9 [51,63] or T10 [6,13,52-54]. Our profiles of cell loss and apoptosis were established from cord injured at vertebra T13, equal to lumber L2 of the cord within the lumbar enlargement. This added new information for a severe injury in the lumber enlargement where the hind limb motoneurons are located. Cell loss in the lumbar enlargement is more related to residual locomotor function than corresponding loss at other sites [62].

Apoptosis is characterized by changes such as cell surface blebbing, cell shrinking, formation of apoptotic bodies, nuclear chromatin condensation, compaction of the cytoplasmic organelles, nuclear lobation and a typical ladder-like electrophoretic pattern of DNA cleaved into nucleosome-size fragments of 180-200 bp or multiples thereof [64]. DNA fragmentation can be observed morphologically by TUNEL staining and quantitated biochemically by *ELISA*. Numerous endogenous agents formed upon initial insult can cause apoptosis through different pathways, many of which converge at the caspase activation cascade [65,66]. Active caspase-3 is the downstream executioner of the caspase cascade [67]. Caspase-3 activation follows SCI [6,68-70]. The discovery that some apoptotic cells do not necessarily go through DNA fragmentation and that cells with DNA fragmentation may die by necrosis led to the use of caspase-3 activation as another indicator of apoptosis. Therefore, in addition to characterizing DNA fragmentation by TUNEL, DNA laddering, and *ELISA*, this study also detected caspase-3 activation to further characterize apoptosis. Although apoptosis has been reported frequently after SCI, as cited in the Background section, few reports have qualitatively and quantitatively studied SCI-induced apoptosis in the lumbar enlargement.

In our previous study, using TUNEL staining as a marker of possible apoptosis, we obtained the temporal and spatial profile of total TUNEL-positive cells. Using TUNEL and NSE double staining, we also established the profile of TUNEL-positive neurons in the lumbar enlargement area after SCI [43]. The present study, further morphologically characterized neuronal and glial apoptosis in the lumbar enlargement using the sections selected at the peak time and distance of apoptosis according to our previously established profiles. TUNEL or active caspase-3 as apoptosis markers were double stained with specific cell markers (NSE, ChAT and GFAP). The TUNEL-positive or active caspase-3-positive neurons, motoneurons and astrocytes in the double-stained sections in the ventral gray matter of injured spinal cords were observed at 24 h (for TUNEL + cell markers) and 12 h (for active fragment p20 of caspase-3 + cell marker) post-SCI (Figures 3 and 4), indicating apoptosis of neurons, motoneurons and astrocytes. Neuronal and glial apoptosis was confirmed by TEM, a gold standard to determine apoptosis based on its specific morphology. Figure 5 illustrates the different stages of neuronal and glial apoptosis by observation of their ultra-structural changes in the ventral gray matter in spinal cord sections removed at 24 h post-SCI, as described in the Results. Motoneuron apoptosis have been reported following compression SCI [69,71], but few reports are available of motoneuron apoptosis after contusion SCI. Our characterization provides important evidence for motoneuron apoptosis after contusion SCI in the lumbar enlargement. Using TUNEL staining for DNA fragmentation and casepase-3 activation as two indicators of cellular apoptosis, our data support the notion that apoptosis is a common mechanism for neurons, motoneurons and glial cells to die following contusion SCI.

Apoptotic DNA fragmentation was also characterized biochemically in the spinal cord tissue by DNA laddering and quantitated by *ELISA* at 48 h (when the maximum number of TUNEL-positive cells appeared) post-SCI. Agarose gel electrophoresis and autoradiography indicated that the intensity of DNA fragment ladders was higher in the epicenter than 1 cm away from the epicenter, and no fragmented DNA bands were observed at 2 cm away from the epicenter or in the sham-operated sections. Compared with the size markers, these DNA fragments were confirmed to have characteristic oligonucleosome-length fragments at intervals of about 180 bp (Figure 6A). *ELISA* quantitation demonstrated that DNA fragmentation decreased with distance from the epicenter. The values in injured animals for each section were 2- to 10-fold that of the corresponding sections in sham-operated rats (Figure 6B). The decline in values over the distance from the epicenter as measured by *ELISA* was comparable to the decline in intensity of DNA laddering over the same distance. The fact that fragmentation was detected in tissue 2 cm from the epicenter and in the sham controls by *ELISA*, but no bands were observed in these samples by electrophoresis reflects the greater sensitivity of *ELISA* over DNA laddering. We also demonstrated that the caspase-3-like protease activity was significantly higher at the epicenter than 1 cm away from the epicenter or in the sham control (Figure 6C). Lack of caspase-3 activity at 1 cm from the epicenter may be attributed to the early measurement of caspase-3 activation at 4 h post-SCI.

Apoptosis is important in regulating normal development and maintaining tissue homeostasis in the adult. However, too much or too little apoptosis may result in pathological disorders. Therefore, there should be a basal level of apoptosis in the normal cord. This basal level was measured in the present study by *ELISA* in the sham-operated cord (Figure 6B). As mentioned in the Methods, to establish the temporal and spatial profile of cellular apoptosis, the TUNEL-positive cells were counted from 0 to 4 mm caudal from the epicenter at 1 h to 1 week post-SCI. At 4 mm, TUNEL positive cells appeared only at 24, 48 and 72 h post-SCI without significant differences from the sham control [43]. The significantly higher level of DNA fragmentation measured by *ELISA* in the injured cord relative to the sham-operated cord was found even at 2 cm caudal from the epicenter. This indicates a greater sensitivity of *ELISA,* impossible by morphological observation of TUNEL-positive cells at such distance. Clearly, morphological examination of TUNEL-positive and caspase-3-positive neurons, motoneurons and astrocytes, and TEM identification are feasible for characterized apoptosis in different types of cells at the cellular level, whereas biochemical analysis of DNA fragmentation and caspase activation has great value in determining the spread of apoptosis from the epicenter. So the results of biochemical analysis provide a great addition to the morphological observation at longer distances from the epicenter.

We previously demonstrated that MnTBAP (i.p.) significantly reduced neuron death at sections 1–2.5 mm rostral and 1 mm caudal from the epicenter [43]. The present study demonstrated that antioxidant treatment with a combination of MnTBAP + L-NA significantly reduced total cell and motoneuron loss in the gray matter of the cord and glial cell loss in the ventromedial white matter of the cord compared with vehicle-treated rats (Figure 7). Significant increases in the number of glial cells in the white matter by the combination treatment was closer to the epicenter (at sections 0 – 1.0 mm from the epicenter) compared with the increases of total cells (at sections 1.5 – 2.5 mm from the epicenter) and motoneurons (at sections 2.0 and 2.5 mm from the epicenter) in the gray matter. This is owing to the fact that most cells near the epicenter in the gray matter of the cord have been lost and cannot benefit from the treatment, whereas the glial loss was much slighter in the ventral white matter, so that many glia are still alive even in the epicenter, to be counted to evaluate the effectiveness of the treatment. Attenuation of total cell and motoneuron loss by the combination suggests that RS contribute to cell death after SCI.

We previously demonstrated that MnTBAP (i.p.) significantly reduced the TUNEL-positive neurons at 1 mm caudal from the epicenter [43]. In the present study, we demonstrated that the combination of MnTBAP + L-NA significantly reduced the numbers of TUNEL-positive cells in the gray and white matter of the cord as compared with the vehicle treatment: an approximately 2.4 times reduction in the gray matter and approximately 10 times reduction in the white matter (Figure 8). The more effective reduction of TUNEL-positive cells in white matter compared with those in gray matter might be attributable to the much slighter cell loss in the ventral white matter as shown in Figure 7. We further demonstrated that MnTBAP alone (i.p.) significantly reduced the number of TUNEL-positive cells in the gray matter of the cord at sections 0, 1 and 2 mm rostral from the epicenter, with the number of TUNEL-positive cells in the vehicle-treated sections approximately 3, 2 and 2.5 times higher than those in the corresponding sections in the MnTBAP-treated group (Figure 9). The reports that treatment with antioxidants, RS scavengers and iron-chelators all reduce RS production, oxidative stress and apoptotic cell death after SCI links RS with apoptotic cell death in SCI [10-13,69,70,72-75]. The impressive reduction in apoptosis by MnTBAP or its combination found in this study strongly supports the notion of the correlation of RS overproduction and apoptosis in SCI and provides further evidence of the causal relationship between RS overproduction and necrotic and apoptotic cell losses. The present results ― together with our previous finding that the combination or MnTBAP alone significantly reduces oxidation and nitration of proteins and MLP, cell death, and neurological deficits after SCI in rats [18,21,38-40,43] ― support the sequence RS → oxidative stress → cell death → neuronal dysfunction.

Using behavioral tests, we recently reported that, with its lower ability than MP to penetrate the BSB, 10 mg/kg MnTBAP administered (i.p.) 4 h post-SCI followed by one half of the first dose 2 h later significantly increased BBB and inclined plane scores compared to vehicle treatment. Post-SCI treatment with MnTBAP is significantly more effective than MP for improving functional recovery after SCI [40]. In the present study, we demonstrated that 15 min pre- and 6 h post-SCI treatment with MnTBAP significantly increased the scores of BBB and beam walk tests, and increased the angle of inclined plane compared with the vehicle-treated group. Our pre- and post-SCI treatment by MnTBAP strongly support the candidacy of MnTBAP for SCI treatment. However, the scores for all treatments in injured animals were significantly worse compared to sham controls (p < 0.001 for all), indicating that pharmaceutical treatments alone cannot improve function to normal levels.

The BBB test is a standard behavioral test for estimating the time course of recovery after SCI and for evaluating the efficiency of treatment. An injury force of 25 g.cm (10 g weight drop 2.5 cm down onto the exposed cord) was popularly used for the contusion injury, and the BBB scores of rats corresponding to this injury force are from 0 to 6 – 10 overtime [6,13,51]. In the present study, the contusion force was 12.5 g.cm and the BBB scores of rats were from 0 to approximately 15 overtime. A similar BBB scores (0 to approximately 14) was reported by Hyun et al. [63] using the 12.5 g.cm injury force on T9. Using the IH device, Springer et al. [54] reported that a 150 kilodyne injury force (which gives an injury comparable to that produced by a 12.5 g.cm weight drop with the NYU device) contused on T10, produced BBB scores of 0 to 12. Apparently, the BBB scores are correlated with the injury force and our results are comparable with other reports with similar injury force. This is also supported by our recent publication in which a 12.5 g.cm injury force was used on T10 [40].

We have demonstrated *in vivo* that RS administered into an uninjured rat spinal cord at a SCI-induced concentration and duration can directly oxidize major cellular components, thereby inducing necrotic and apoptotic cell death and neurological deficits, and that the removal of RS by MnTBAP significantly reduces the RS-induced damage [27-31] - directly and unequivocally demonstrating RS-induced oxidation to the major cellular components as an important pathway for cell death after SCI. Since the oxidative defense enzymes (such as superoxide dismutase, catalase, glutathione peroxidase, thioredoxin and others) are all susceptible to RS-induced oxidative damage, they may suffer function-distorting oxidative modification, in turn causing more RS production to form a negative feedback amplification of the oxidative damage. MnTBAP scavenges SCI-induced RS, protecting against oxidative damage to these enzymes and thereby restoring their enzymatic activities. This process reverses the negative to positive feedback amplification. RS can also cause injury by acting as intracellular death signals that lead to changes in the expression of proteins. RS may be active in different cell death pathways, including the caspase activation cascade. Therefore the removal of RS by MnTBAP reduces apoptotic cell death. RS can also act as modulators of the redox state, and the right dose of MnTBAP treatment to remove overproduced RS helps to maintain the redox balance, thereby avoiding further damage. Whether RS are the initiators or the intracellular messengers in cell death pathways after CNS injury, MnTBAP provides an opportunity for treatment by removing RS. In addition to catalytically scavenging a wide range of RS, other mechanisms may also contribute to the beneficial effects of metalloporphyrins, such as modulating RS-based redox signaling pathways and regulating cellular transcription activity [33]. It has been reported that MnTBAP attenuated the nuclear translocation of apoptosis-inducing factor and the subsequent DNA fragmentation induced by ROS after permanent focal cerebral ischemia in mice [76]. Therefore, in addition to its enzymatic catalytic scavenging activities, more complicated mechanisms may be involved in our *in vivo* finding that MnTBAP reduced cell death including apoptosis and improved functional recovery after SCI.

## Conclusion

1) By counting the surviving cells in the spinal cord sections at the distances of 0 to 4 mm caudal from the epicenter and at different times from 1 h to 1 week post-SCI, the present study established the temporal and spatial profiles of motoneuron and glial cell loss following a severe experimental SCI (50 g.cm) at the lumbar enlargement area. The loss of both motoneuron and glial cells is time- and distance-dependent. The number of surviving motoneurons decreased over time and increased over distance from the epicenter. In contrast, glia loss in the ventral white matter is much slighter and more limited at the epicenter and quickly offset over time by proliferation. Our three dimensional profiles of the different types of cell loss provide a database for determining the effective time and location of therapeutic intervention. 2) By counting surviving cells in the spinal cord sections at the distances from 0 to 4 mm rostral from the epicenter, the present study established the spatial profiles of MnTBAP + L-NA protection against different types of cell loss at the lumbar enlargement area following SCI. MnTBAP + L-NA significantly increased the number of surviving cells at 1.5 – 2.5 mm, surviving motoneurons at 2 and 2.5 mm in the gray matter, and surviving glial cells at 0 – 1 mm in the white matter rostral from the epicenter. The effectiveness of this combination treatment links cell death with SCI-induced RS elevation. 3) Apoptosis of neurons, motoneurons and astrocytes was characterized in injured spinal cord sections morphologically by double immuno-staining and double immuno-fluorescence-staining with TUNEL or active caspase-3 + specific cell markers. Neuronal and glial apoptosis was confirmed by TEM identification. Our results, consistent with other reports, suggest that apoptosis is a common mechanism for different types of cell death after SCI. Using DNA laddering, *ELISA* and caspase-3 activation in spinal cord tissue at different distances from the epicenter, we demonstrated that apoptotic DNA fragmentation and caspase-3 activation declined with the distance from the epicenter. *ELISA* quantitation indicated that significant DNA fragmentation in the injured cords extended over 2 cm caudal to the epicenter compared with the sham control cords. 4) By counting TUNEL-positive cells in the spinal cord sections, we explored the protective ability of both MnTBAP alone and its combination with L-NA against apoptotic cell death after SCI. MnTBAP + L-NA significantly reduced the number of TUNEL-positive cells with an approximately 2.4-fold reduction in the ventral gray matter and an approximately 10-fold reduction in the ventral white matter. The combination seems more effective in reducing the number of TUNEL-positive cells than in increasing the number of surviving cells. MnTBAP alone significantly reduced the number of TUNEL-positive cells at 0, 1 and 2 mm rostral from the epicenter to approximately 3, 2 and 2.5 times lower than in corresponding sections of the vehicle-treated group. 5) MnTBAP protection against neurological deficits was evaluated by behavioral tests for 9 weeks. We demonstrated for the first time that 15 min pre- and 4 h post-SCI treatment with MnTBAP (10 mg/kg, i.p.) significantly increased the scores on BBB and beam walk tests, and increased the angle of the inclined plane test compared to vehicle-treated groups, demonstrating that antioxidant therapy with MnTBAP significantly improved functional recovery compared with vehicle treatment. This is a most important criterion for determining the therapeutic potential of a candidate agent for SCI treatment. Our demonstration that apoptotic cell death follows SCI and that MnTBAP, alone or in combination, significantly reduced apoptosis correlates SCI-induced apoptosis with RS overproduction. It provides evidence that the generation of RS during the secondary damage cascade after SCI is a cardinal factor leading to secondary neuronal and glial death, thereby causing neurological dysfunction. Therefore, antioxidant therapy is likely to play an important role in the future treatment of SCI. Our results suggest that MnTBAP is a potential candidate for antioxidant therapy following SCI.

## Abbreviations

ANOVA: Analysis of variance; BBB-test: Basso-Beattie-Bresnahan locomotor rating scale; BSB: Blood–spinal cord barrier; ChAT: Choline acetyltransferase; CHAPS: 3-[(3-cholamidopropyl) dimethylammonio]-1-propane-sulfonate; CNS: Central nervous system; CV: Cresyl violet; ELISA: Enzyme-linked immuno-sorbent assay; TEM: Transmission electron microscope; FITC: Fluorescein isothiocyanate; GFAP: Glial fibrillary acidic protein; HEPES: 4-(2-hydroxyethyl)-1-piperazineethanesulfonic acid; i.p.: Intraperitoneally; i.v.: Intravenously; L-NA: Nitro-L-arginine; MLP: Membrane lipid peroxidation; MnTBAP: Mn (III) tetrakis (4-benzoic acid) porphyrin; MP: Methylprednisolone; MPSS: Methylprednisolone sodium succinate; ●NO: Nitric oxide; NOS: Nitric oxide synthase; NSE: Neuron-specific enolase; NYU: New York University; PBS: Phosphate-buffered saline; RMANOVA: Repeated measures of analysis of variance; RS: Reactive species; s.c.: Subcutaneously; SCI: Spinal cord injury; SD: Standard deviation; SEM: Standard error of the mean; TRITC: Crystalline tetramethylrhodamine isothiocyanate; TUNEL: Terminal deoxynucleotidyl transferase (TdT) mediated deoxyuridine triphosphate-(dUTP)-biotin nick end labeling.

## Competing interests

No competing financial or non-financial interests exist for any of the authors.

## Authors’ contributions

DL, the Principal Investigator, conceived the study, designed the experiments, trained the postdoctoral fellows and oversaw their work, analyzed and developed the presentation of the data, participated in the experiments of triple blinded behavioral tests, and wrote the entire manuscript. XL, a postdoctoral fellow, carried out all experiments to establish the temporal and spatial profiles of cell loss, the spatial profile of MnTBAP and the combination of MnTBAP + L-NA protection against cell loss and apoptosis; participated in morphologically characterizing apoptosis by immuno-staining and biochemical analysis of caspase-3 activation, and in determining MnTBAP protection against neurological dysfunction following experimental SCI; prepared figures; performed statistical analysis of data; and jointly prepared the portion of the manuscript pertaining to her contribution to the research. FB, a postdoctoral fellow, carried out the experiments for TEM confirmation of apoptosis; participated in morphologically characterizing apoptosis by immuno-staining; and in the experiment of behavioral tests for MnTBAP protection against neurological dysfunction following SCI; prepared figures; performed statistical analysis of data; and jointly helped prepare the portion of the manuscript pertaining to his contribution to the research. HQ, a postdoctoral fellow, carried out the experiment of DNA laddering, and jointly helped prepare the portion of the manuscript pertaining to his contribution. All authors read and approved the final manuscript.
